# The molecular basis of ADP-ribose pyrophosphorylation by a phage PRPS-like enzyme in NAD^+^ reconstitution

**DOI:** 10.1093/nar/gkag901

**Published:** 2026-09-18

**Authors:** Mengxi Liu, Jingbo Ma, Qingqing Zhang, Shaoting Xu, Jing Chen, En Lin, Yunkun Wu, Hua Li, Biao Zhou, Lixia Chen

**Affiliations:** Institute of Digital Materia Medica and Structural Pharmacology, Fujian Key Laboratory of Chinese Materia Medica, College of Pharmacy, Fujian University of Traditional Chinese Medicine, Fuzhou 350122, China; State Key Laboratory of Respiratory Disease, Guangzhou Chest Hospital, Institute of Tuberculosis, Guangzhou Medical University, Guangzhou 510095, China; Wuya College of Innovation, Key Laboratory of Structure-Based Drug Design & Discovery, Ministry of Education, Shenyang Pharmaceutical University, Shenyang 110016, China; Wuya College of Innovation, Key Laboratory of Structure-Based Drug Design & Discovery, Ministry of Education, Shenyang Pharmaceutical University, Shenyang 110016, China; Wuya College of Innovation, Key Laboratory of Structure-Based Drug Design & Discovery, Ministry of Education, Shenyang Pharmaceutical University, Shenyang 110016, China; Wuya College of Innovation, Key Laboratory of Structure-Based Drug Design & Discovery, Ministry of Education, Shenyang Pharmaceutical University, Shenyang 110016, China; Institute of Digital Materia Medica and Structural Pharmacology, Fujian Key Laboratory of Chinese Materia Medica, College of Pharmacy, Fujian University of Traditional Chinese Medicine, Fuzhou 350122, China; Institute of Digital Materia Medica and Structural Pharmacology, Fujian Key Laboratory of Chinese Materia Medica, College of Pharmacy, Fujian University of Traditional Chinese Medicine, Fuzhou 350122, China; State Key Laboratory of Respiratory Disease, Guangzhou Chest Hospital, Institute of Tuberculosis, Guangzhou Medical University, Guangzhou 510095, China; Institute of Digital Materia Medica and Structural Pharmacology, Fujian Key Laboratory of Chinese Materia Medica, College of Pharmacy, Fujian University of Traditional Chinese Medicine, Fuzhou 350122, China; Wuya College of Innovation, Key Laboratory of Structure-Based Drug Design & Discovery, Ministry of Education, Shenyang Pharmaceutical University, Shenyang 110016, China

## Abstract

Depletion of cellular NAD^+^ is an increasingly recognized bacterial antiphage strategy, and many phages counter this pressure by rebuilding NAD^+^ from its cleavage products. In the NARP1 pathway, Adps converts ADP-ribose (ADPR) and ATP into ADPR-PP, the immediate substrate for Namat-dependent NAD^+^ synthesis, but how a phosphoribosyl pyrophosphate synthetase (PRPS)-like fold catalyzes this noncanonical reaction has been unknown. Here we report structures of phage Adps in apo, ADPR/ATP-bound pre-catalytic, and ADPR-PP/AMP-bound product states, supported by LC-MS activity assays, kinetics, and mutagenesis. The structures show that Adps uses a conserved interdomain groove to bind the ADPR acceptor, while a remodeled PP loop and catalytic β-hairpin create a donor site that positions the ATP β–γ pyrophosphate next to the distal ribose of ADPR. Lys185 moves toward the reaction center during the transition from substrate to product states, and its substitution abolishes ADPR-PP formation and impairs Adps–Namat-mediated rescue. Comparison with canonical PRPS enzymes indicates that Adps retained an ancestral acceptor-recognition surface while rewiring donor binding and regulatory elements. These findings define the molecular basis of phage ADPR pyrophosphorylation and illustrate how viral enzymes can repurpose conserved nucleotide-metabolic scaffolds to restore NAD^+^ during host immune attack.

## Graphical Abstract

**Graphical Abstract:**
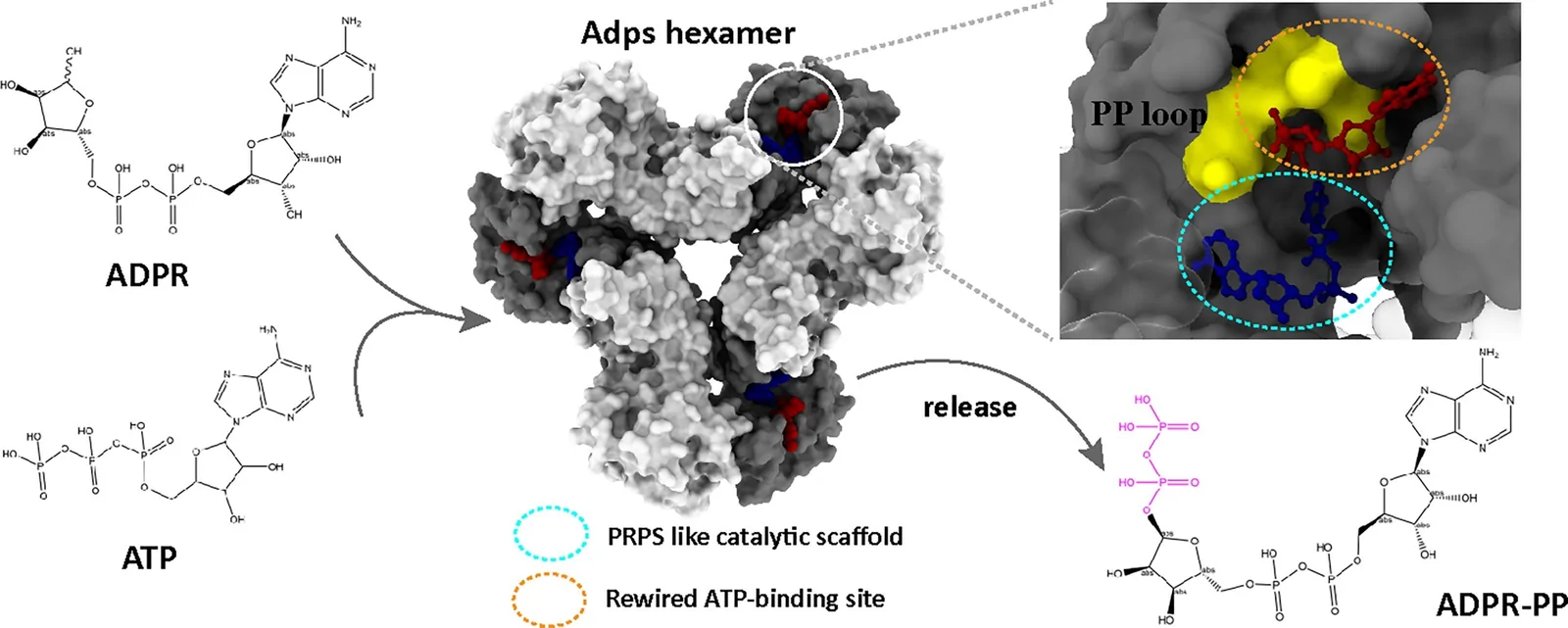

## Introduction

The conflict between bacteria and bacteriophages has produced a wide range of defense and counter-defense strategies [1–3]. Bacterial immunity includes restriction-modification, CRISPR-Cas, and many recently discovered abortive-infection systems [4–8]. A recurring theme in these systems is the targeted disruption of central metabolites, including NAD^+^ and nucleotide-derived molecules [9–14]. In several antiphage pathways, TIR- or Sir2-domain enzymes are activated after phage detection and deplete NAD^+^ to ADP-ribose (ADPR) and nicotinamide (NAM), thereby limiting the metabolic capacity required for productive infection [15, 16]. Confirmed NADase-dependent systems include DSR2 [16–18], SPARSA [19], Sir2-HerA [20–22], Kongming [23–25], SPARTA [19], TIR-CARF [26], and TIR-STING modules in CBASS [27]. Thus, NAD^+^ metabolism is not only a housekeeping process but also a major battleground in phage–host immunity.

Phages have evolved countermeasures that restore the NAD^+^ pool after immune activation. Phylogenetic and biochemical analyses identified two phage NAD^+^ reconstitution pathways (NARP1 and NARP2) [28]. NARP1 encodes Adps (ADP-ribose pyrophosphorylase) and Namat (nicotinamide ADPR transferase). Adps converts ADPR into ADP-ribose pyrophosphate (ADPR-PP), and Namat then condenses ADPR-PP with NAM to regenerate NAD^+^ [28]. In NARP2, Adps is absent and Namat instead uses NAM and phosphoribosyl pyrophosphate (PRPP) to produce nicotinamide mononucleotide, which is subsequently converted to NAD^+^ [28]. Approximately 4.3% of sequenced bacteriophages encode NARP1 [28], suggesting that ADPR-PP formation is a recurring phage solution to NAD^+^-depleting immunity. Adps is annotated as a member of the phosphoribosyl pyrophosphate synthetase (PRPS) family, whose canonical members synthesize PRPP from ribose-5-phosphate (R5P) and ATP and thereby support purine, pyrimidine, tryptophan, and NAD^+^ biosynthesis [29–31]. Adps presents a mechanistically unusual example of PRPS-like chemistry. Like PRPS, it uses ATP as a pyrophosphate donor and adopts a PRPS-like catalytic scaffold, but its acceptor is ADPR, a larger and chemically distinct nucleotide-derived metabolite. The product, ADPR-PP, is not a standard intermediate of cellular metabolism and acts as the committed substrate for NARP1-dependent NAD^+^ rebuilding. How a PRPS-like fold accommodates ADPR and redirects pyrophosphate transfer to this noncanonical acceptor has remained unknown [28].

Three mechanistic features are therefore central to understanding Adps catalysis. First, the enzyme must recognize the adenine, phosphoribose, and distal ribose elements of ADPR within a scaffold related to enzymes that normally bind R5P. Second, ATP must be positioned so that its β–γ pyrophosphate is delivered to the distal ribose of ADPR rather than to the canonical PRPS acceptor. Third, canonical PRPS enzymes are controlled by allosteric nucleotides and, in several systems, by higher-order assembly [32, 33]. Whether phage Adps retains these regulatory features or has been streamlined for rapid NAD^+^ reconstitution was unclear.

Here, we combine X-ray crystallography, cryo-EM, and biochemical analysis to define three functional states of Adps: apo-Adps at 2.61 Å, a pre-catalytic ADPR/ATP-bound state at 2.76 Å, and a post-catalytic ADPR-PP/AMP-bound state at 2.98 Å. These structures, together with LC-MS product detection, steady-state kinetics, mutational analysis, and comparison with canonical PRPS catalytic sites, explain how Adps binds ADPR, positions ATP for pyrophosphate transfer, and uses Lys185 as a key catalytic residue. Comparisons with canonical PRPS enzymes further show that Adps preserves an ancestral acceptor-recognition surface while rewiring donor binding and discarding major PRPS regulatory/assembly modules. The work provides a molecular framework for NARP1 and illustrates a general route by which phage enzymes can refactor conserved nucleotide-metabolic folds to counter host immunity.

## Materials and methods

### Adps expression and purification

The codon-optimized gene encoding Adps (UniProt: F1D1Q1) was cloned into pET-28a to generate an N-terminal 6× His-tagged fusion construct. The recombinant plasmid was transformed into *Escherichia coli* Rosetta (DE3) cells. Cells were grown in LB medium at 37°C to an OD_600_ of 0.8–1.0, induced with 0.2 mM isopropyl β-D-1-thiogalactopyranoside (IPTG), and incubated at 16°C for 12 h. Cell pellets were resuspended in ice-cold lysis buffer (20 mM Tris–HCl, pH 8.5, 150 mM NaCl, 5 mM β-mercaptoethanol, and 1 mM phenylmethylsulfonyl fluoride (PMSF)), disrupted by sonication and clarified by centrifugation. The supernatant was loaded onto a Ni-NTA column and washed extensively before elution with 300 mM imidazole in 50 mM Tris–HCl, pH 8.5, 150 mM NaCl, and 1 mM PMSF. The eluate was further purified by size-exclusion chromatography on a Superdex 200 Increase 10/300 GL column (Cytiva) equilibrated in buffer D (20 mM Tris–HCl, pH 7.5, 150 mM NaCl, and 1 mM dithiothreitol (DTT)). Fractions containing Adps were pooled, concentrated, and stored at −80°C.

### Crystallization, X-ray data collection, and structure determination

Crystals of apo-Adps were obtained by hanging-drop vapor diffusion at 16°C after screening commercial and homemade sparse-matrix conditions with a Mosquito crystallization robot (SPT Labtech). Diffraction-quality crystals grew from 0.1 M Bis-Tris, pH 6.5, and 25% (w/v) polyethylene glycol 3350 using protein at ~12 mg/ml. Crystals were cryoprotected with 20% (v/v) glycerol and flash-cooled in liquid nitrogen. X-ray diffraction data were collected at beamline BL-02U1 of the Shanghai Synchrotron Radiation Facility and processed with XIA2 and CCP4. The structure was solved by molecular replacement using the AlphaFold3 model as the search model, manually rebuilt in COOT [34], and refined with REFMAC in CCP4.

### Cryo-EM sample preparation and data collection

Purified Adps was concentrated to ~5 mg/ml immediately before grid preparation. For preparation of the ligand-bound complexes, MgCl_2_ was added to a final concentration of 5 mM. To prepare the pre-catalytic complex, purified Adps was mixed with ADPR and ATP at a molar ratio of 1:2:2 (Adps:ADPR:ATP), and the mixture was immediately used for cryo-EM grid preparation to minimize turnover and capture the substrate-bound state. To prepare the post-catalytic complex, an identical Adps–ADPR–ATP mixture was incubated for 1 h before grid preparation, allowing the reaction to proceed and the product-bound ADPR-PP/AMP state to form. For each sample, 3 μl was applied to glow-discharged Quantifoil Au R1.2/1.3 300-mesh grids. Grids were blotted for 2.5 s at 4°C and 100% humidity with a blot force of 5 and plunge-frozen in liquid ethane using a Vitrobot Mark IV (Thermo Fisher Scientific).

All cryo-EM datasets were collected on a Titan Krios G4 microscope (Thermo Fisher Scientific) operated at 300 kV and equipped with a Falcon 4 direct electron detector and a Selectris X energy filter set to a 10 eV slit width. Automated acquisition was performed using EPU software at a nominal magnification of 165 000×, corresponding to a calibrated pixel size of 0.73 Å/pixel. Micrographs were recorded over a defocus range of −0.8 to −2.4 µm, with a total accumulated exposure of ∼50 e^−^/Å^2^ per micrograph.

### Cryo-EM data processing

Cryo-EM data for the Adps–ADPR–ATP and Adps–ADPR–PP-AMP complexes were processed in cryoSPARC [35] unless otherwise stated. Movies were corrected for beam-induced motion and subjected to patch CTF estimation [36]. Micrographs with poor CTF fits, excessive drift, crystalline ice, or obvious contamination were removed manually. After curation, 3283 micrographs were retained for the Adps–ADPR–ATP dataset and 2815 micrographs for the Adps–ADPR–PP-AMP dataset.

For the Adps–ADPR–ATP dataset, particles were first identified by blob picking, and representative particles were used to generate templates for template picking. A total of 995 129 particle images were extracted and subjected to iterative 2D classification. Classes with clear Adps features yielded 202 037 particles for *ab initio* reconstruction. Reference-free models were then used for heterogeneous refinement in C1 symmetry to remove incomplete, poorly aligned, and contaminating particles. A well-resolved hexameric class containing 82 500 particles was selected for refinement with D3 symmetry.

For the Adps–ADPR–PP-AMP dataset, blob-based and template-based particle picking yielded 160 157 particle images. Following 2D classification, particles with clear structural features were used for *ab initio* reconstruction. Multiple initial models were generated to capture compositional and conformational heterogeneity and were subsequently used as references for heterogeneous refinement in C1 symmetry. The major intact hexameric classes were combined, and 66 000 particles were retained for high-resolution refinement without imposed symmetry.

The selected particle sets were subjected to non-uniform refinement followed by iterative global and local CTF refinement. The final Adps–ADPR–ATP and Adps–ADPR–PP-AMP maps reached overall resolutions of 2.76 and 2.98 Å, respectively, based on the gold-standard Fourier shell correlation criterion of 0.143. Local-resolution distributions were estimated in cryoSPARC.

### Model building, refinement, and validation

The apo-Adps model was docked into the cryo-EM maps of the ligand-bound complexes using UCSF Chimera [37]. Models were manually adjusted in COOT [34] and refined in real space with PHENIX [38] using secondary-structure and geometry restraints. Model quality was assessed with the validation tools in PHENIX.

### The catalytic activity assay of Adps

Purified Adps and variants were assayed in 200 μl reactions containing 20 mM Tris–HCl, pH 8.0, 150 mM NaCl, 0.5 mM ADPR, 2.5 mM ATP, 12 mM MgCl_2_, 1 mM DTT, and 50 μg purified protein. Reactions were incubated at 30°C for 1 h with shaking at 200 rpm and quenched by adding 100 μl methanol. Products were analyzed by LC-MS.

Chromatographic separation was performed on a Vanquish Flex UHPLC system (Thermo Fisher Scientific, Germering, Germany) using a Waters ACQUITY UPLC HSS T3 column (50 × 2.1 mm, 1.8 μm). Mobile phase A was 10 mM ammonium formate in water, and mobile phase B was 10 mM ammonium formate in 95:5 (v/v) methanol:water. The flow rate was 0.35 ml/min, and the gradient was 5% B for 0–0.5 min, 40% B at 2 min, 100% B at 8–10 min, and 5% B at 10.1–12 min. The injection volume was 2 μl. The UHPLC was coupled to an Orbitrap Exploris 120 mass spectrometer (Thermo Fisher Scientific, Dreieich, Germany) equipped with a heated electrospray ionization source operated in negative mode. Source parameters were as follows: spray voltage, −2500 V; sheath, auxiliary, and sweep gas flow rates, 50, 10, and 1 arbitrary units, respectively; capillary temperature, 325°C; vaporizer temperature, 350°C. All assays were performed in three independent experiments and yielded consistent results.

### Magnesium ion dependency assay

The effect of Mg^2+^ concentration on Adps activity was evaluated under the same reaction conditions described earlier. Briefly, purified protein was incubated in a 25 μl reaction mixture containing 20 mM Tris–HCl (pH 8.0), 150 mM NaCl, 0.5 mM ADPR, 1 mM ATP, and varying concentrations of MgCl_2_ (0, 0.5, 1, 2, 5, 10, and 20 mM). Reactions containing 0 mM MgCl_2_ were used as the reference condition, and enzyme activity under this condition was defined as 100%.

To evaluate the metal ion dependency, control reactions were performed in the presence of 20 mM ethylenediaminetetraacetic acid (EDTA) or 20 mM CaCl_2_ instead of MgCl_2_. After incubation under identical reaction conditions, AMP production was quantified using the AMP-Glo™ assay. Each condition was analyzed using independent replicates, and data are presented as mean values ± standard deviation (SD) (*n* = 5).

### AMP inhibition assay

Purified Adps were subjected to AMP sensitivity analysis in a 25 μl reaction system containing 20 mM Tris–HCl (pH 8.0), 150 mM NaCl, 0.15 mM ADPR, 0.1 mM ATP, 12 mM MgCl_2_, and 0.025 μM purified protein. AMP was supplemented at final concentrations of 0, 0.05, 0.1, 0.25, and 0.5 mM. For each AMP concentration, a corresponding AMP-only control reaction containing the same amount of AMP but lacking purified protein was prepared to account for background AMP-derived signals.

Following completion of the endpoint reactions, AMP production was quantified using the AMP-Glo™ assay. The AMP-dependent signal contribution was determined by subtracting the luminescence signal obtained from the AMP-only controls from that of the corresponding enzymatic reactions. Each AMP concentration was analyzed using five independent biological replicates. Data presented are shown as mean values ± SD (*n* = 5).

### Steady-state kinetic analysis

To measure the *k*_cat_ and Michaelis constant, *K*_m_, values, experiments were carried out with the same reagents and buffer as above. Values were calculated by varying ADPR concentration (0.02–5 mM) with a constant ATP concentration (2 mM) or by varying ATP concentration (0.02–6 mM) with a constant ADPR concentration (2 mM). Adps concentration in these experiments was 0.025 μM, and the reaction was incubated for 10 min at 25°C and stopped by reagent 1 (AMP-Glo™ assay).

### Bacterial growth curve assay

Bacterial growth assays were used to evaluate whether Adps–Namat counteracts the growth defect associated with DSR2 and TTP expression. Cells carrying the indicated plasmid combinations were cultured overnight and diluted into fresh medium under the appropriate antibiotic selection and induction conditions. The tested conditions were a control strain, DSR2 plus TTP, Adps–Namat with DSR2 plus TTP, and the catalytic mutant Adps (K185A)–Namat with DSR2 plus TTP. Cultures were incubated at 37°C with shaking, and OD_600_ was measured at 0, 2, 4, 6, 8, 10, and 12 h. Growth curves were plotted to compare the effects of DSR2 and the indicated phage counter-defense components.

## Results

### Apo-Adps forms a PRPS-like hexamer

**Figure 1.**
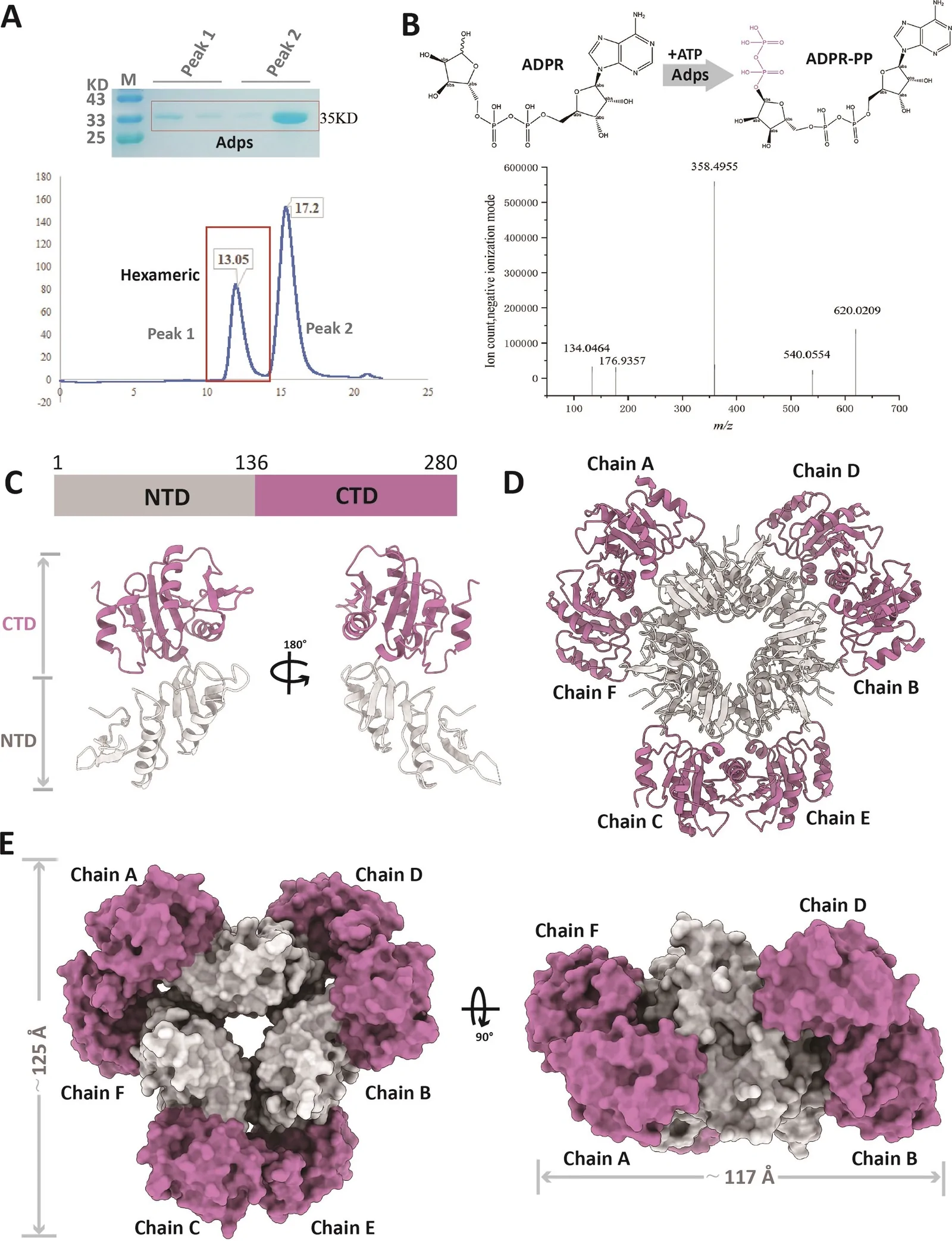
Crystal structure of apo-Adps. (**A**) Size-exclusion chromatography profile of purified Adps. Two elution peaks were observed and analyzed by sodium dodecyl sulfate–polyacrylamide gel electrophoresis. The major band migrated at ~35 kDa, consistent with the expected molecular weight of monomeric Adps. The earlier elution peak, corresponding to an apparent hexameric species, is highlighted by the red box. (**B**) LC-MS analysis of the reaction containing Adps, ADPR, and ATP. The extracted ion chromatogram and mass spectrum support formation of ADPR-PP. (**C**) Domain organization of an Adps subunit. The N-terminal domain (NTD, residues 1–136) is shown in gray, and the C-terminal domain (CTD, residues 137–280) in purple. (**D**) Overall apo-Adps hexamer, shown as a ring-like trimer of dimers. (**E**) Surface view of the Adps hexamer from top and side orientations, illustrating the approximate dimensions and subunit packing.

Initial expression of Adps from bacteriophage SpβL1 yielded largely insoluble protein, consistent with previous work [28]. We therefore selected an Adps homolog from Vibrio phage ICP1, which could be purified by Ni^2+^-affinity chromatography followed by size-exclusion chromatography. SEC revealed two peaks eluting at 13.05 and 17.20 ml, indicating that purified Adps possesses distinct oligomeric states in solution (Fig. 1A). LC-MS confirmed that the purified enzyme converted ADPR and ATP into ADPR-PP, as reported for SpβL1 Adps [28], establishing that the ICP1 homolog retains ADPR pyrophosphorylation activity (Fig. 1B).

To understand how Adps catalyzes ATP and ADPR into ADPR-PP, we determined the crystal structure of apo-Adps (Supplementary Table S1). Crystals diffracted to 2.61 Å, and the structure was solved by molecular replacement using an AlphaFold3 model as the search template [39]. The asymmetric unit contains one Adps subunit composed of two domains. The N-terminal domain (residues 1–136) contains three alpha-helices packed against seven parallel beta-strands. The C-terminal domain (residues 137–282) contains five alpha-helices surrounding four parallel beta-strands and includes a short antiparallel β-hairpin adjacent to one side of the domain (Fig. 1C).

Crystal packing and symmetry analysis show that Adps assembles as a homohexamer with a trimer-of-dimers organization (Fig. 1D). The hexamer forms a compact, crown-like particle of ~125 Å by 117 Å (Fig. 1E). The C-terminal domains occupy the outer rim, whereas the N-terminal domains converge to form the central core. Although Adps catalyzes a reaction different from that of bacterial and eukaryotic PRPS enzymes, its overall hexameric architecture is closely related to canonical PRPS assemblies [32, 33, 40].

### Pre-catalytic structure reveals coordinated recognition of ADPR and ATP

**Figure 2.**
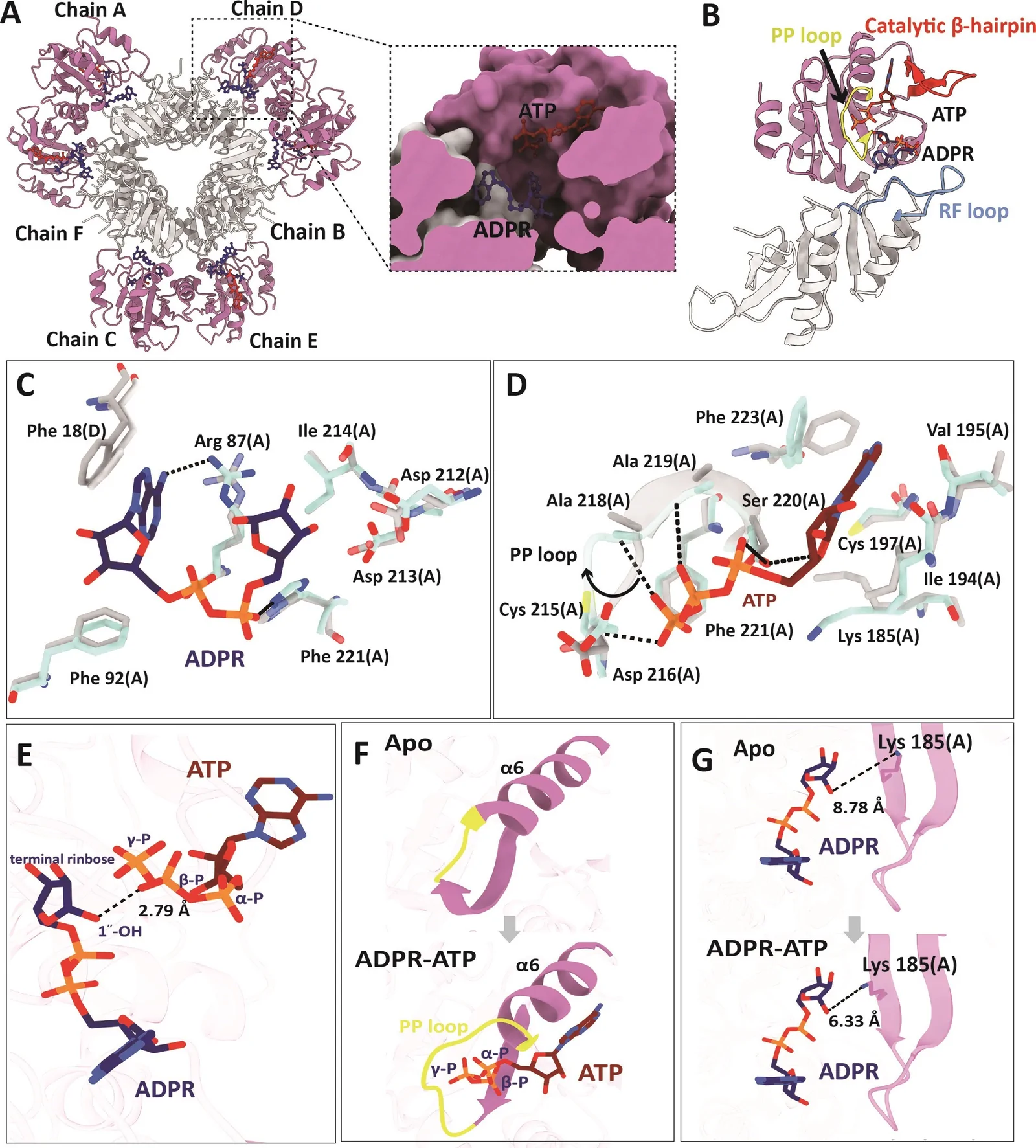
Coordinated recognition of ADPR and ATP by Adps. (**A**) Overall structure of the hexameric Adps–ADPR–ATP complex. The six subunits are designated chains A–F, and the bound ADPR and ATP molecules are shown as sticks. The enlarged surface representation of chain D shows that ADPR and ATP occupy an interdomain pocket. (**B**) Overall structure of an Adps protomer in the ADPR/ATP-bound state, highlighting the PP loop (yellow), catalytic β-hairpin (red), and RF loop (light blue) surrounding the active site. ADPR and ATP are shown as sticks. (**C, D**) Comparison of the substrate-recognition residues between apo-Adps and the ADPR/ATP-bound complex. Panel C shows the residues involved in ADPR recognition, whereas panel D shows the PP loop and neighboring residues involved in ATP recognition. Residues in the ADPR/ATP-bound complex are shown in light cyan, and the corresponding residues in apo-Adps are shown in gray. ADPR and ATP are shown as dark-blue and red sticks, respectively. Dashed lines indicate putative polar interactions, and chain identifiers are given in parentheses. The arrow in panel D indicates ligand-induced repositioning of the PP loop toward the ATP phosphate chain. (**E**) Spatial arrangement of ADPR and ATP in the substrate-bound complex. The putative nucleophilic O1″ atom of the terminal ribose of ADPR is positioned ~2.79 Å from the β-phosphorus of ATP, placing the β–γ pyrophosphoryl group in a configuration compatible with transfer. The α-, β-, and γ-phosphates of ATP are indicated. (**F**) Comparison of the α6/PP-loop region between apo-Adps and the ADPR/ATP-bound complex. Binding of ADPR and ATP induces repositioning of the PP loop toward the ATP phosphate chain. (**G**) Ligand-dependent movement of Lys185 on the catalytic β-hairpin. The distance between Lys185 and the reaction center decreases from ~8.78 Å in apo-Adps to 6.33 Å in the ADPR/ATP-bound complex, positioning Lys185 closer to the distal ribose of ADPR and the ATP phosphate chain.

To capture the substrate-bound state, purified Adps was mixed with ADPR and ATP at a molar ratio of 1:2:2 (Adps:ADPR:ATP) in the presence of MgCl_2_, and the mixture was immediately used for cryo-EM grid preparation. This approach minimized the reaction time before vitrification and enabled us to determine a cryo-EM structure of the Adps–ADPR–ATP complex representing a pre-catalytic state (Supplementary Fig. S1 and Supplementary Table S2). In the cryo-EM structure of Adps determined in the presence of ADPR and ATP, Adps exists as a hexamer, which is similar to the crystal structure of apo Adps (Fig. 1D and E). As expected, continuous non-protein density was observed in the cleft formed by the two domains within each Adps moieties (Fig. 2A). The high resolution allows us to assign the densities to ADPR and ATP (Supplementary Fig. S2). The catalytic pocket is enclosed by the diphosphate loop (PP loop, Cys215 to Ser220), a β-hairpin (Ala183 to Leu 199), and a regulatory flexible loop (RF loop, Leu80 to Pro99), which together form a chamber that accommodates ADPR and the ATP (Fig. 2B).

ADPR occupies a deep groove at the active-site cleft. The terminal ribose is positioned toward the triphosphate of ATP moiety through contacts involving Asp212, Asp213, and the backbone of Ile214. The adenine-containing portion of ADPR is stabilized by Phe18 from the neighboring protomer, together with Arg87 and Phe92 from the principal active-site protomer. Phe18 and Phe92 provide aromatic contacts, whereas Arg87 contributes a polar and/or cation–π interaction (Fig. 2C). This arrangement shows that Adps recognizes ADPR by extending a conserved PRPS-like acceptor-recognition surface rather than by creating a separate, newly evolved acceptor pocket [29, 33].

Adjacent to ADPR, the ATP molecule is sandwiched between the β-hairpin and the α6 helix of Adps, with its phosphate groups oriented toward the ribose of ADPR. The triphosphate backbone is coordinated by Asp216, Ala218, Ala219, and Ser220, which form a phosphate-recognition network expected to stabilize the negatively charged donor and the developing transition state. The ribose and adenine portions of the donor are supported by hydrophobic contacts with Ile194, Val195, Cys197, and Cys215, whereas Phe221 and Phe223 form a lid-like cap over the adenine base (Fig. 2D). Together, the two substrates are arranged so that the β–γ pyrophosphate lies immediately next to the distal ribose of ADPR, creating the geometry needed for pyrophosphate transfer (Fig. 2E).

Substrate binding causes only modest changes in the overall hexamer. The largest rearrangements are local and occur around the PP loop. In particular, the C-terminal end of α6 partially unwinds and becomes more loop-like, expanding the donor-binding pocket relative to apo-Adps (Fig. 2F). This remodeling allows ATP accommodation without disrupting the PRPS-like catalytic core.

The β-hairpin that surround ATP also undergoes a clear substrate-induced shift. In apo-Adps, the side chain of Lys185 points away from the reaction center. In the ADPR/ATP-bound structure, it moves inward toward the distal ribose of ADPR (Fig. 2G). This movement couples ligand binding to active-site organization and suggested that Lys185 might contribute directly to pyrophosphate transfer.

### Adps repurposes a PRPS-like scaffold by rewiring donor binding

**Figure 3.**
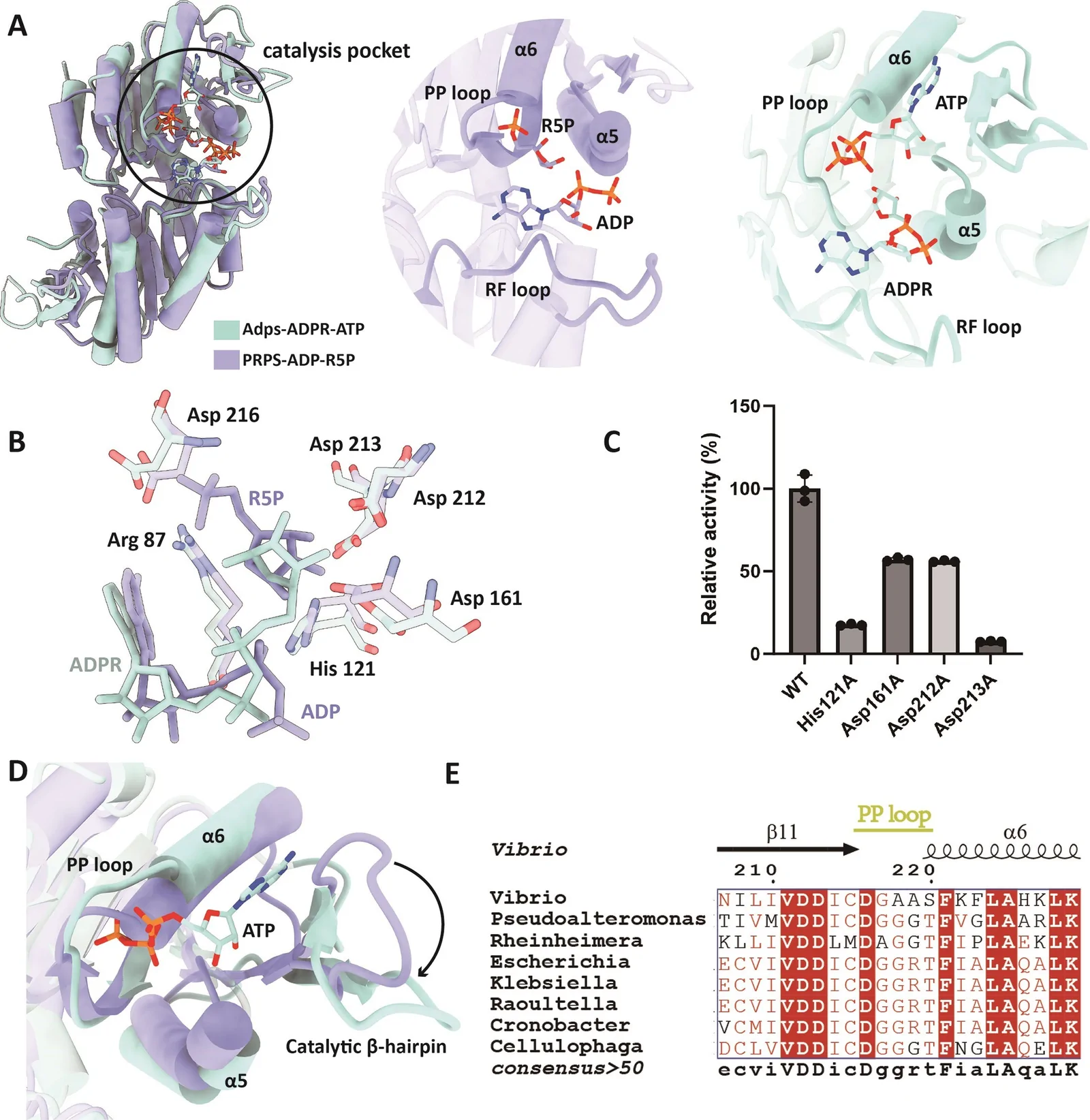
Evolutionary repurposing of a PRPS-like scaffold for ADPR pyrophosphorylation.(**A**) Structural comparison of the Adps–ADPR–ATP complex (cyan) with human PRPS1 bound to ADP and R5P (purple; PDB: 8DBE). The overall superposition is shown on the left, with the catalytic pocket indicated by a circle. Enlarged views show the corresponding ligand-binding pockets of PRPS1 and Adps. (**B**) Superposition of the acceptor-binding regions of Adps and PRPS1. ADPR in Adps spans the regions corresponding to the ADP- and R5P-binding sites of PRPS1(PDB: 8DBE). Conserved or structurally equivalent residues implicated in substrate recognition, including Arg87, His121, Asp161, Asp212, Asp213, and Asp216, are shown as sticks. (**C**) Relative ADPR-PP-forming activities of wild-type Adps and the His121A, Asp161/A, Asp212A, and Asp213A variants. Activities were normalized to that of wild-type Adps, which was set to 100%. Data are shown as mean ± SD from three independently prepared reactions. (**D**) Local remodeling of the PP loop and neighboring α6 region creates an ATP-binding mode distinct from canonical PRPS(PDB:8DBE). (**E**) Multiple sequence alignment of representative Adps homologs showing conservation of the PP-loop region involved in donor recognition.

To enable a comparative analysis of the structural features distinguishing Adps from PRPS, we superimposed the Adps–ADPR–ATP complex with the canonical PRPS structures (Fig. 3A). While the core folds of Adps and PRPS are similar, the substrate–binding pockets for ADPR/ATP in Adps are distinct from the ADP/R5P–binding sites in PRPS. In Adps, it can be observed that ADPR occupies the position corresponding to the ADP and R5P sites of PRPS: the adenine and proximal ribose overlap with ADP, whereas the distal ribose occupies the position of R5P of PRPS. Several residues are implicated in substrate recognition, including Arg87, His121, Asp161, Asp212, Asp213, and Asp216, which are conserved in position (Fig. 3B). Unlike to PRPS, where these residues play an essential role in coordinating ADP and R5P, in Adps, they stabilize different portions of ADPR. Alanine substitution of residues in this pocket reduced ADPR-PP formation to different extents under the standard assay conditions (Fig. 3C). Consistent with these activity measurements, steady-state kinetic analysis showed that all four variants exhibited increased apparent *K*_m_ values and reduced apparent catalytic efficiencies toward both ADPR and ATP compared with wild-type Adps (Supplementary Fig. S3).

As for the ATP–binding region in Adps, it differs substantially from the equivalent region in canonical PRPS. In PRPS, the PP loop helps position the phosphate of R5P and the pyrophosphate-transfer machinery around the canonical acceptor. In Adps, this region is repurposed to engage the ATP-derived pyrophosphate donor directly. The PP loop and neighboring α6/catalytic β-hairpin region remodel to create a donor-binding geometry that places the β–γ pyrophosphate next to the distal ribose of ADPR (Fig. 3D). Thus, Adps appears to have acquired ADPR pyrophosphorylation not by inventing a new acceptor site, but by reusing a pre-existing acceptor-recognition surface and changing how ATP is bound and aligned.

Multiple sequence alignment shows that the PP-loop region involved in donor recognition is conserved across representative Adps homologs (Fig. 3E and Supplementary Fig. S4). This conservation suggests that donor-site repurposing is a family-wide feature rather than a property of a single phage enzyme. More broadly, the comparison provides a sequence-structure rule for annotating PRPS-like phage proteins that may participate in NAD^+^ reconstitution.

### Product-state structure defines the pyrophosphate-transfer trajectory

**Figure 4.**
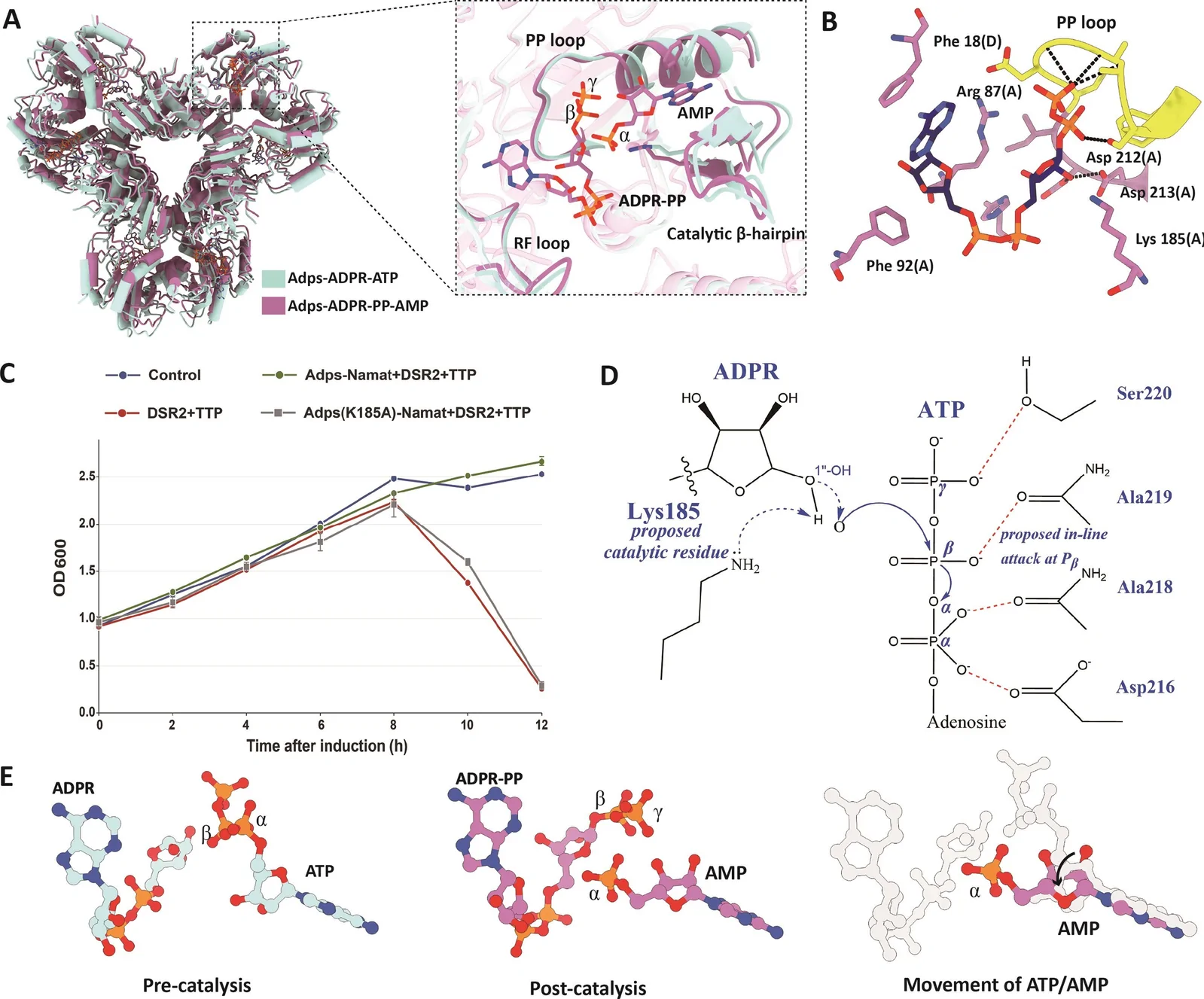
Structural and mechanistic basis of Adps-catalyzed pyrophosphate transfer. (**A**) Superposition of the pre-catalytic Adps–ADPR–ATP complex (cyan) and post-catalytic Adps–ADPR-PP–AMP complex (magenta). The enlarged active-site view highlights the relative positions of the PP loop, RF loop, and catalytic β-hairpin, together with the substrate- and product-state ligands. The α-, β-, and γ-phosphates of ATP and the corresponding product-state ligands are indicated. (**B**) Recognition of ADPR-PP in the post-catalytic state. Phe18 and Phe92 contribute to recognition of the adenine-containing portion of ADPR-PP, whereas Arg87, Asp212, Asp213, Lys185, and the PP loop surround the distal ribose and newly transferred pyrophosphate group. Dashed lines indicate putative polar interactions. Chain identifiers are shown in parentheses. (**C**) Growth analysis of cells expressing the indicated defense and counter-defense components. OD_600_ was monitored for 12 h after induction for the control, DSR2 plus TTP, Adps–Namat plus DSR2/TTP, and Adps (Lys185A)–Namat plus DSR2/TTP conditions. (**D**) Proposed electron-pair flow mechanism for Adps-catalyzed ADPR pyrophosphorylation. The terminal-ribose O1″ of ADPR is proposed to attack the β-phosphorus of ATP, accompanied by cleavage of the Pβ–Oα bond and transfer of the β,γ-diphosphoryl group to ADPR. Lys185 is positioned to assist nucleophile alignment and activation and/or to stabilize charge development at the reaction center; whether it directly functions as a general base remains unresolved. The PP-loop region, including the backbone amides of Ala218, Ala219, and Ser220, contributes to organization of the ATP phosphate chain. Blue arrows indicate the proposed electron-pair movements, and red dashed lines indicate putative phosphate-positioning interactions. Mg^2+^ is not depicted because no discrete metal-binding site could be assigned in the cryo-EM map. (**E**) Comparison of the pre-catalytic ADPR/ATP-bound and post-catalytic ADPR-PP/AMP-bound states.

To understand how Adps recognizes the reaction products and how the active site reorganizes after pyrophosphate transfer, we sought to capture Adps in a product-bound state. Purified Adps was mixed with ADPR and ATP at a molar ratio of 1:2:2 and incubated for 1 h before cryo-EM grid preparation, allowing ADPR-PP and AMP to form. This yielded a 2.98 Å cryo-EM structure of the Adps–ADPR–PP-AMP complex (Supplementary Figs S5 and S6). Superposition with the pre-catalytic Adps–ADPR–ATP structure shows that the hexameric assembly and overall fold remain largely unchanged (Fig. 4A). The reaction therefore appears to proceed through localized active-site rearrangements rather than through a global conformational transition.

In the product complex, ADPR-PP occupies the same interdomain groove used by ADPR. The adenine ring remains anchored by Phe18, Arg87, and Phe92, and the newly formed pyrophosphate is coordinated by the PP loop (Fig. 4B). Comparison of substrate- and product-bound states reveals a progressive inward movement of Lys185 on the catalytic β-hairpin (Supplementary Fig. S7A and B). In the product state, the Lys185 side chain approaches both the distal ribose and the nascent pyrophosphate, a geometry consistent with a role in orienting the nucleophile and stabilizing negative charge during transfer. We thus hypothesize that Lys185 is a key enzymatically active residue in Adps, similar to the role of human PRPS1, which transfers the pyrophosphate group from ATP to R5P to yield PRPP and AMP. In PRPS1, Lys194 in the catalytic loop forms hydrogen–bonding interactions with the ATP β–phosphate, implicating it directly in phosphate–transfer [33]. This is comparable to Lys185 in the β–hairpin region of Adps; moreover, sequence alignment shows that the basic residue equivalent to Lys185 is conserved across representative PRPS homologs (Supplementary Fig. S7C).

We next tested whether Lys185 is required for Adps activity. LC-MS analysis showed robust ADPR-PP formation by wild-type Adps, whereas no product was detected for the K185A variant under the same assay conditions (Supplementary Fig. S8). We further assessed the functional consequence of this substitution using the DSR2/TTP growth assay, in which activation of the DSR2 NADase by the phage tail tube protein causes NAD^+^ depletion [18]. Wild-type Adps–Namat substantially restored bacterial growth, whereas Adps(K185A)–Namat failed to provide comparable protection and phenocopied the DSR2/TTP condition at late time points (Fig. 4C). Thus, the K185A substitution abolishes detectable enzymatic activity and compromises NARP1-mediated counter-defense, supporting an essential role for Lys185 in Adps catalysis.

These observations support a working model for Adps-catalyzed pyrophosphoryl transfer (Fig. 4D). ADPR and ATP are first preorganized in adjacent acceptor- and donor-binding regions. Lys185 then moves toward the reaction center and is proposed to assist positioning and activation of the terminal-ribose O1″ for attack on the ATP β-phosphorus. The PP loop organizes the ATP phosphate chain and may stabilize charge development during cleavage of the Pβ–Oα bond, producing ADPR-PP and AMP. After transfer, rotation of the AMP adenosine moiety may facilitate product release (Fig. 4E). The present structures and mutational data do not establish whether Lys185 directly acts as a general base; substrate polarization, electrostatic stabilization, or a water-mediated proton-transfer mechanism remain possible. Nevertheless, its ligand-dependent movement, structural correspondence to the catalytic-loop lysine of PRPS1, evolutionary conservation, and K185A loss-of-function phenotype together identify Lys185 as a key catalytic-site residue in Adps.

### Adps is uncoupled from canonical PRPS regulation

**Figure 5.**
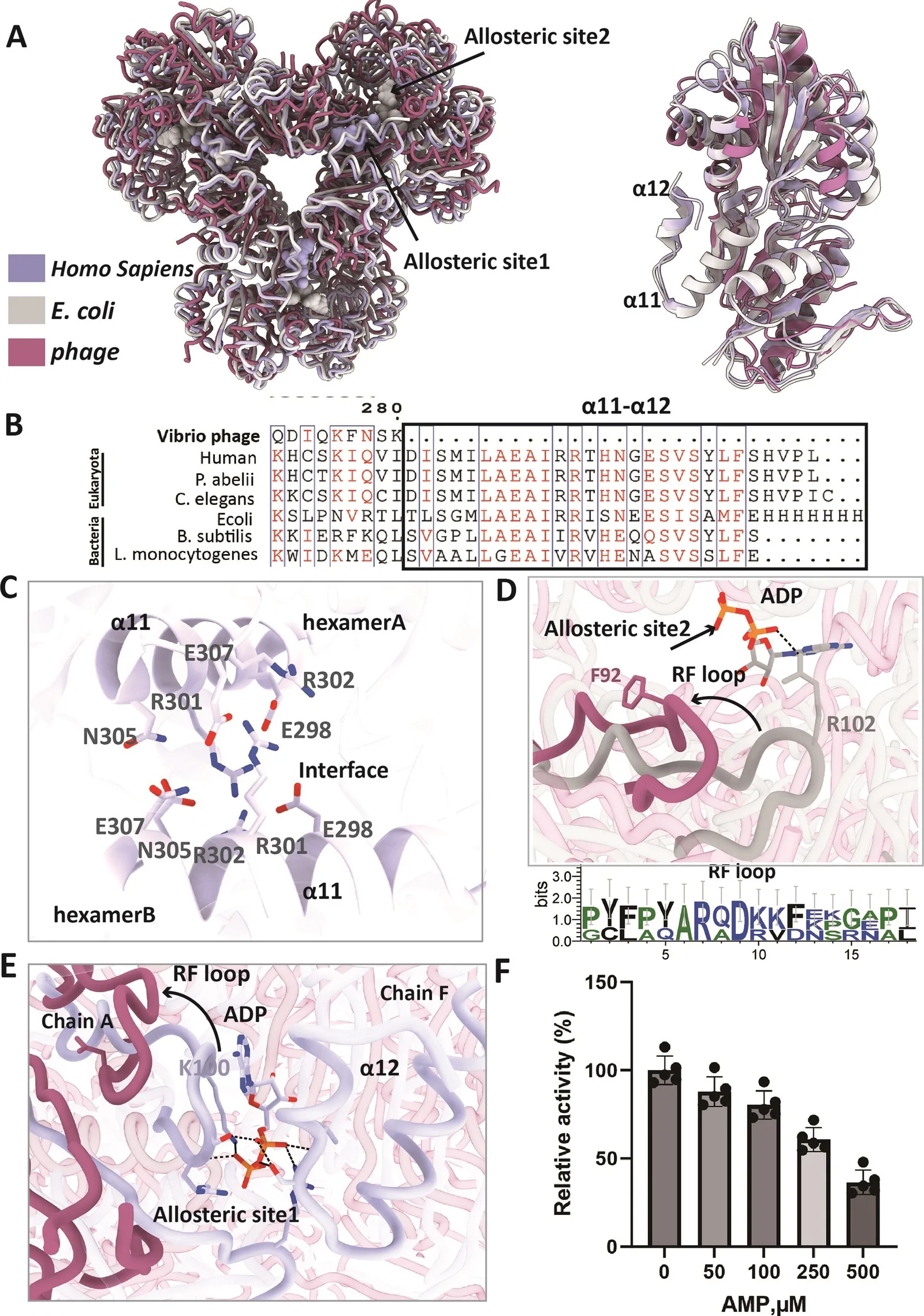
Structural divergence uncouples Adps from canonical PRPS filamentation and allosteric regulation. (**A**) Structural superposition of the phage Adps monomer (this study) with PRPS orthologs from *Homo sapiens* (PDB:8DBE) and *E. coli* (PDB:7XMU). Global comparison of phage Adps with human and bacterial PRPS homologs, highlighting classical allosteric sites and the α11–α12 module in PRPS. (**B**) Sequence alignment showing loss of the C-terminal α11–α12 region in Adps homologs. (**C**) PRPS filament-forming interface mediated by α11–α12 and associated inter-subunit contacts. (**D**) Degeneration of the canonical allosteric site 2 in Adps, including RF-loop remodeling and loss of conserved ADP-coordinating residues. (**E**) Remodeling of the region corresponding to allosteric site 1. (**F**) Effect of AMP on Adps activity. Relative activity was measured in the presence of 0, 50, 100, 250, or 500 μM AMP and normalized to the no-AMP control. Bars represent the mean ± SD of five independently prepared reactions, and individual measurements are shown as black circles.

Adps retains the PRPS catalytic core and hexameric assembly, but comparison with bacterial and eukaryotic PRPS enzymes reveals substantial divergence in regions associated with higher-order assembly and allosteric regulation (Fig. 5A). These differences suggest that the phage enzyme has been streamlined for a metabolic counter-defense role rather than preserved as a fully regulated PRPS enzyme.

One major difference is the absence of the C-terminal α11–α12 module in Adps (Fig. 5A and B). Multiple sequence alignment indicates that this region is consistently missing from Adps homologs (Fig. 5B), suggesting that this structural truncation represents a defining evolutionary signature of the Adps family. In human and bacterial PRPS structures, α11–α12 contributes to filament-forming interfaces through inter-subunit contacts involving residues such as Arg301, Arg302, Glu307, and Asn305 [32, 33, 41] (Fig. 5C). Loss of this module explains why Adps cannot form the same PRPS-type filament interface, although it does not exclude the possibility of other higher-order assemblies formed by different surfaces.

Beyond the assembly interfaces, the canonical allosteric regulatory sites in PRPS have undergone profound degeneration in Adps. The canonical PRPS allosteric sites are also poorly conserved. In PRPS, allosteric site 2 binds ADP and communicates inhibitory signals through the RF loop [32]. In Adps, the corresponding RF loop adopts a different conformation, and the residues that coordinate ADP in PRPS are not conserved (Fig. 5D). Allosteric site 1, which lies at an inter-subunit interface in PRPS, is likewise remodeled in Adps (Fig. 5E). These structural changes are not compatible with the conserved ADP-binding pockets that regulate canonical PRPS activity.Thus, Adps preserves the PRPS catalytic framework but has lost or remodeled key regulatory and filamentation elements. Such streamlining would be advantageous for a phage enzyme that must rapidly convert immune-generated ADPR into ADPR-PP during NAD^+^ reconstitution. The Adps family therefore illustrates how viral enzymes can retain the chemistry of a host-like metabolic fold while discarding regulatory features that are unnecessary, or potentially limiting, in the context of counter-defense.

In addition, we independently examined whether the reaction product AMP inhibits Adps. Exogenous AMP caused a concentration-dependent decrease in enzyme activity over the range of 50–500 μM, with ~35% residual activity observed at 500 μM AMP (Fig. 5F). These results demonstrate that AMP inhibits Adps under the conditions tested, independently of the canonical PRPS allosteric regulatory architecture. However, whether AMP inhibits Adps through product inhibition at the catalytic site or by binding to a distinct regulatory site remains to be further investigated.

## Discussion

Large-scale discovery and mechanistic characterization of antiphage systems have revealed that bacterial immunity frequently targets central metabolism as well as genetic material [7, 42]. NAD^+^ is a prominent example. Several defense systems activate TIR- or Sir2-related effectors that deplete NAD^+^ or convert it into immune signaling molecules, thereby restricting phage propagation [18, 20, 26, 43]. Other systems use NAD^+^ as a cofactor for DNA ADP-ribosylation or assemble NADase-active complexes after recognition of phage-associated nucleic acids or signaling molecules [41, 44, 45]. These examples place NAD^+^ and ADPR metabolism at the center of phage–host conflict and raise the question of how phages restore the metabolites consumed by host defense.

Phages can sense and exploit host-derived signals, as illustrated by quorum-sensing-like systems in SPβ and vibriophage VP882 [46–49]. NARP1 extends this concept from signal sensing to metabolite repair: Adps and Namat together convert the products of NAD^+^ cleavage, ADPR and NAM, back into NAD^+^ [28]. The structure of Namat has recently explained how the pathway recognizes ADPR-PP [50]. Our work defines the upstream step, showing how Adps produces ADPR-PP from ADPR and ATP. The two enzyme structures now provide a continuous molecular explanation for NARP1-mediated NAD^+^ rebuilding.

A central insight from this study is that Adps generates a noncanonical nucleotide-derived metabolite by refactoring, rather than replacing, a conserved enzyme scaffold. The ADPR acceptor is accommodated on a PRPS-like surface that already contains elements for adenine, ribose, and phosphate recognition. The major innovation lies in the donor site: the PP loop and catalytic β-hairpin are reorganized to position ATP for transfer of the β–γ pyrophosphate to the distal ribose of ADPR. This division of labor—conserved acceptor recognition combined with redesigned donor positioning—offers a parsimonious route for the emergence of new nucleotide-metabolic reactions.

A closer comparison with canonical PRPS enzymes further clarifies how Adps has altered an otherwise highly conserved catalytic architecture. In canonical PRPS [29, 40], catalysis is organized around the coordinated recognition of ATP, R5P, and the activating phosphate. The active site therefore integrates two chemically distinct substrates within a relatively compact pocket, with the R5P-binding region and ATP-binding region positioned to support transfer of the β–γ pyrophosphate group of ATP to the 1′-hydroxyl of R5P. Structural studies of bacterial and eukaryotic PRPS have shown that this catalytic arrangement is highly conserved despite substantial differences in regulation and higher-order assembly [29, 32, 33, 40]. The Adps structure reveals that this conservation extends beyond the overall fold: the adenine-, ribose-, and phosphate-recognition elements used by canonical PRPS have been retained sufficiently to accommodate ADPR. Rather than replacing the ancestral acceptor-recognition architecture, Adps extends it to a larger substrate in which the adenine-containing portion and proximal ribose occupy positions analogous to those of ADP, whereas the distal ribose is placed in the region normally occupied by R5P. This arrangement provides a mechanistic explanation for why a PRPS-like scaffold can tolerate an apparently radical change in substrate specificity. The evolutionary innovation therefore appears to have occurred primarily by reinterpreting the spatial relationships among pre-existing recognition elements rather than by constructing an entirely new acceptor-binding pocket.

The comparison also highlights a more fundamental distinction in the handling of the pyrophosphate donor. In canonical PRPS, the acceptor R5P is positioned so that its reactive hydroxyl group approaches the ATP β-phosphate, while the surrounding loops organize the ATP phosphates and stabilize the transition state. In Adps, the distal ribose of ADPR occupies an acceptor position analogous to R5P, but the additional adenine and ribose-phosphate groups of ADPR constrain how ATP can approach the reaction center. Our pre-catalytic structure shows that the PP loop and neighboring α6 region undergo local remodeling, generating a donor-binding configuration in which the ATP β–γ pyrophosphate is rotated and translated toward the distal ribose of ADPR. Thus, the difference between PRPS and Adps is not simply a change in the chemical identity of the acceptor. It is a coordinated rewiring of substrate geometry in which the acceptor-recognition surface remains largely ancestral, whereas the donor-recognition machinery is reconfigured to accommodate the altered topology of ADPR. This distinction may explain why relatively localized sequence and structural changes can generate a new enzymatic activity while preserving the overall PRPS fold.

In canonical PRPS, Mg^2+^ is essential for ATP-dependent pyrophosphate transfer, where metal coordination to the ATP phosphate groups helps organize the triphosphate chain and position the β-phosphate for nucleophilic attack [29, 33]. Our biochemical analysis similarly shows that Adps activity is dependent on Mg^2+^, whereas substitution with Ca^2+^ or chelation by EDTA does not support the same catalytic state (Supplementary Fig. S9). However, since no discrete Mg^2+^ density could be assigned unambiguously in the cryo-EM maps, precluding a definitive description of its coordination geometry, its exact structural role remains to be fully elucidated. Given that the PP loop, α6 region, and catalytic β-hairpin are reorganized to create a donor-binding mode distinct from that of canonical PRPS, Mg^2+^ may act primarily through coordination of the Mg^2+^–ATP complex and/or stabilization of the ATP phosphate chain, while the surrounding protein architecture positions the donor and acceptor for transfer. Thus, Adps may have preserved the metal-dependent chemical constraint of the ancestral PRPS reaction while rewiring the local structural environment in which Mg^2+^ participates. Determining whether Adps retains a PRPS-like metal-coordination mode or has evolved a distinct one will require structures with better-resolved metal-binding states and complementary biochemical analysis.

Comparison with PRPS homologs also suggests that Adps has undergone coordinated changes outside the catalytic center. Canonical PRPS enzymes are not simply constitutive pyrophosphate-transfer enzymes; their activities are tightly integrated with cellular nucleotide metabolism through allosteric regulation and, in many organisms, reversible higher-order assembly. *Escherichia coli* PRPS, e.g. can form distinct filamentous states whose ligand-dependent conformations alter the RF loop and modulate allosteric regulation, while human PRPS1 filaments stabilize allosteric sites and couple polymerization to catalytic control [32, 33]. In these enzymes, therefore, the hexameric state represents only one level of organization, with filament formation providing an additional regulatory layer rather than merely serving as a structural assembly state. Earlier structural studies of human PRPS1 further established that distinct phosphate- and nucleotide-binding sites communicate with the catalytic pocket through flexible-loop rearrangements [40]. These observations are important when interpreting the architecture of Adps: retention of the PRPS-like hexamer should not be taken to mean retention of the canonical PRPS regulatory system.

Indeed, our structural comparison indicates that Adps selectively preserves the oligomeric architecture required to maintain the catalytic scaffold while eliminating the specific structural elements that couple PRPS activity to filamentation and allosteric control. The absence of the C-terminal α11–α12 module is particularly informative because this region participates in the inter-subunit contacts that stabilize PRPS filaments [32, 33]. At the same time, the RF loop and the regions corresponding to the canonical allosteric sites are substantially remodeled in Adps. Thus, the evolutionary trajectory of Adps appears to involve neither wholesale loss of oligomerization nor wholesale preservation of PRPS regulation. Instead, the enzyme retains the compact hexameric framework but disconnects it from the regulatory and assembly mechanisms characteristic of canonical PRPS. This distinction is especially relevant in the context of phage metabolism, where the selective advantage may come from maintaining a stable and catalytically competent oligomer while avoiding regulatory constraints that evolved to balance PRPP production with the broader metabolic state of the host cell.

In summary, our data define the structural basis of ADPR pyrophosphorylation by Adps and explain how this reaction supplies the committed ADPR-PP intermediate for NARP1. The work connects a phage-encoded PRPS-like enzyme to NAD^+^ repair and provides a framework for understanding how viral enzymes diversify nucleotide metabolism during phage–bacteria arms races. Future work that captures short-lived catalytic intermediates, quantifies pathway flux during infection, and compares diverse Adps homologs will further test how broadly this repurposing mechanism operates.

## Conclusion

This study defines how the phage-encoded enzyme Adps converts ADPR and ATP into ADPR-PP, the committed intermediate of the NARP1 NAD^+^ reconstitution pathway. Three structural states, together with biochemical and mutational data, show that Adps retains a PRPS-like acceptor-recognition framework while remodeling the ATP donor site and losing major canonical PRPS regulatory features. These findings explain how a phage enzyme repurposes a conserved nucleotide-metabolic scaffold to counter NAD^+^-depleting immunity. They also provide a general model for viral metabolic innovation, in which new pathway intermediates arise through targeted rewiring of pre-existing enzyme architectures.

## Supplementary Material

gkag901_Supplemental_File

## Data Availability

The atomic coordinates and structure factors for apo-Adps have been deposited in the Protein Data Bank and are available at https://doi.org/10.2210/pdb23bm/pdb. Atomic coordinates for the pre-catalytic Adps–ADPR–ATP complex and the post-catalytic Adps–ADPR–PP-AMP complex have been deposited in the Protein Data Bank and are available at https://doi.org/10.2210/pdb27fn/pdb and https://doi.org/10.2210/pdb23ca/pdb, respectively. Cryo-EM maps for the Adps–ADPR–ATP and Adps–ADPR–PP-AMP complexes have been deposited in the Electron Microscopy Data Bank under accession codes EMD-81126 and EMD-68853, respectively.
