# Supplementary material for: The molecular basis of ADP-ribose pyrophosphorylation by a phage PRPS-like enzyme in NAD+ reconstitution
Source: Nucleic Acids Res. 2026 Sep 18;54(17):gkag901. doi: 10.1093/nar/gkag901 (PMC13586617; doi:10.1093/nar/gkag901)
Supplement: Supplementary file 1 [file gkag901_supplemental_file.pdf]

Supporting Figures

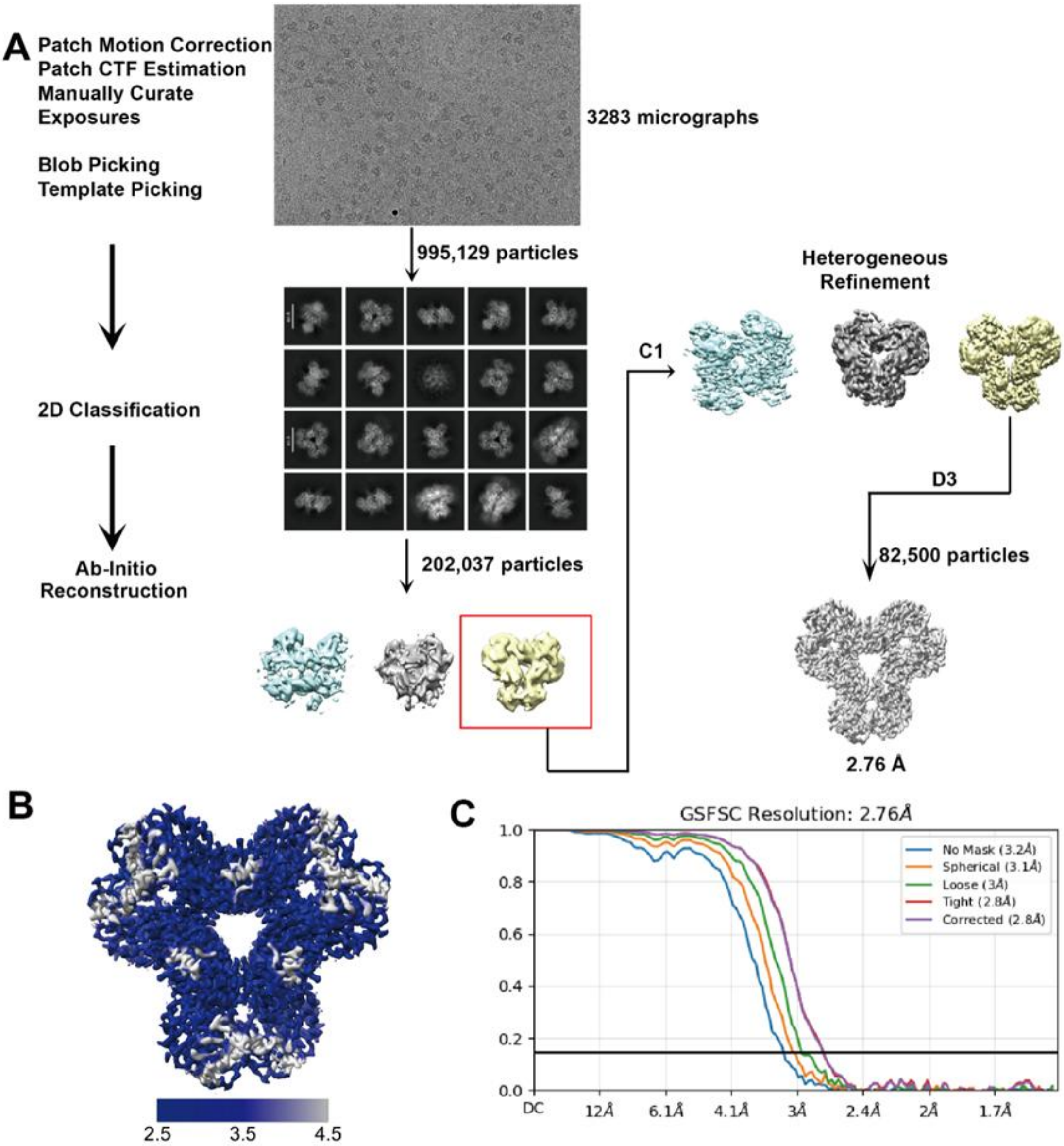

Supplementary Fig. S1. Cryo-EM data processing and validation of the Adps-ADPR-ATP complex. (A) Workflow for micrograph curation, particle picking, 2D classification, ab initio reconstruction, heterogeneous refinement and final 3D refinement of the pre-catalytic Adps complex. Representative micrograph and 2D class averages are shown. **(B)** Final cryo-EM reconstruction colored by local resolution. **(C)** Gold-standard Fourier shell correlation (FSC) curves for the final map and model-to-map validation.

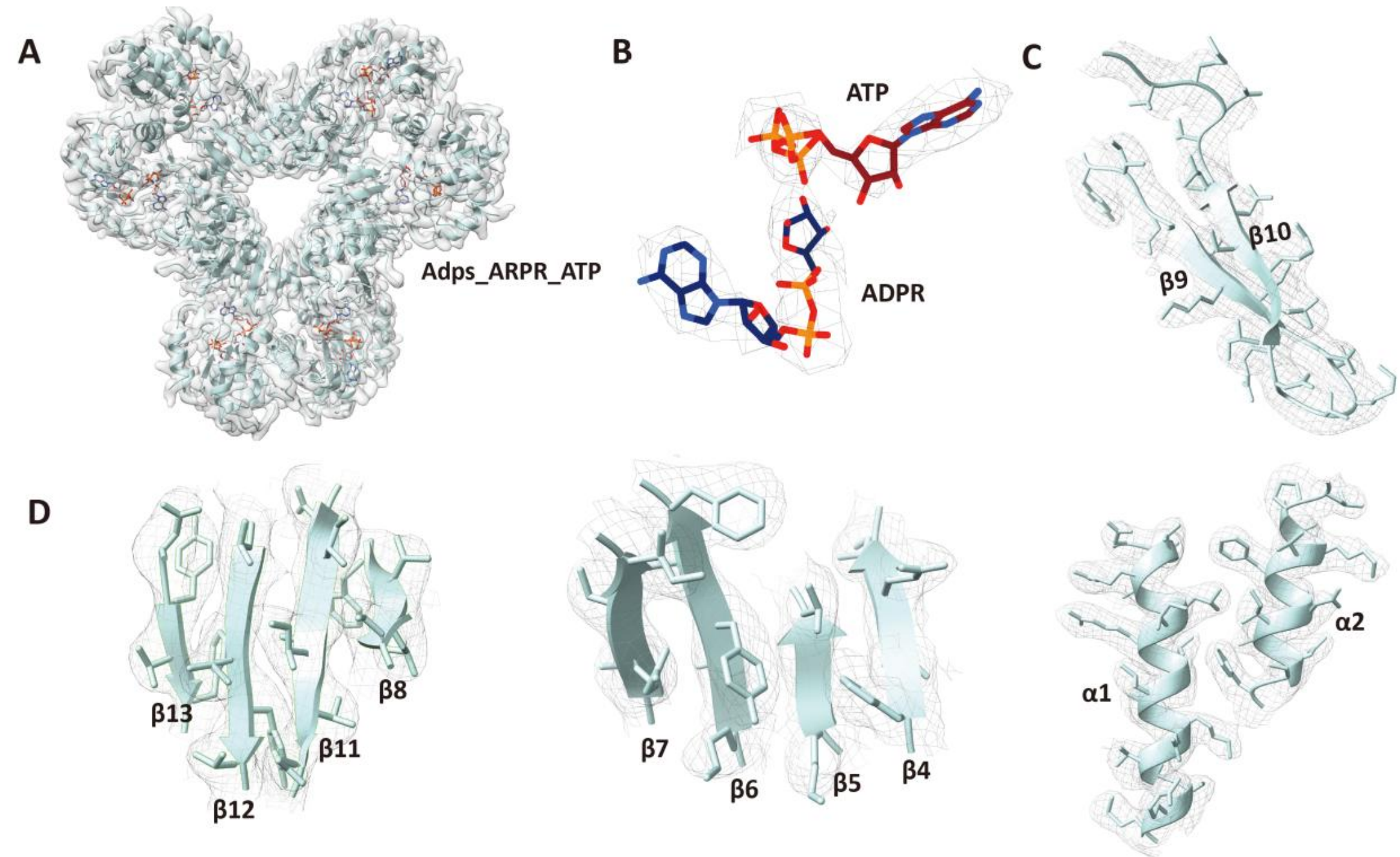

Supplementary Fig. S2. Representative cryo-EM density and model quality for Adps-ADPR-ATP structures. **(A)** Overall cryo-EM maps with fitted atomic models for the ligand-bound Adps hexamers. **(B)** Representative ligand densities supporting assignment of ADPR/ATP. **(C and D)** Representative local density around selected beta-strands and alpha-helical regions, illustrating the quality of model fitting in both complexes.

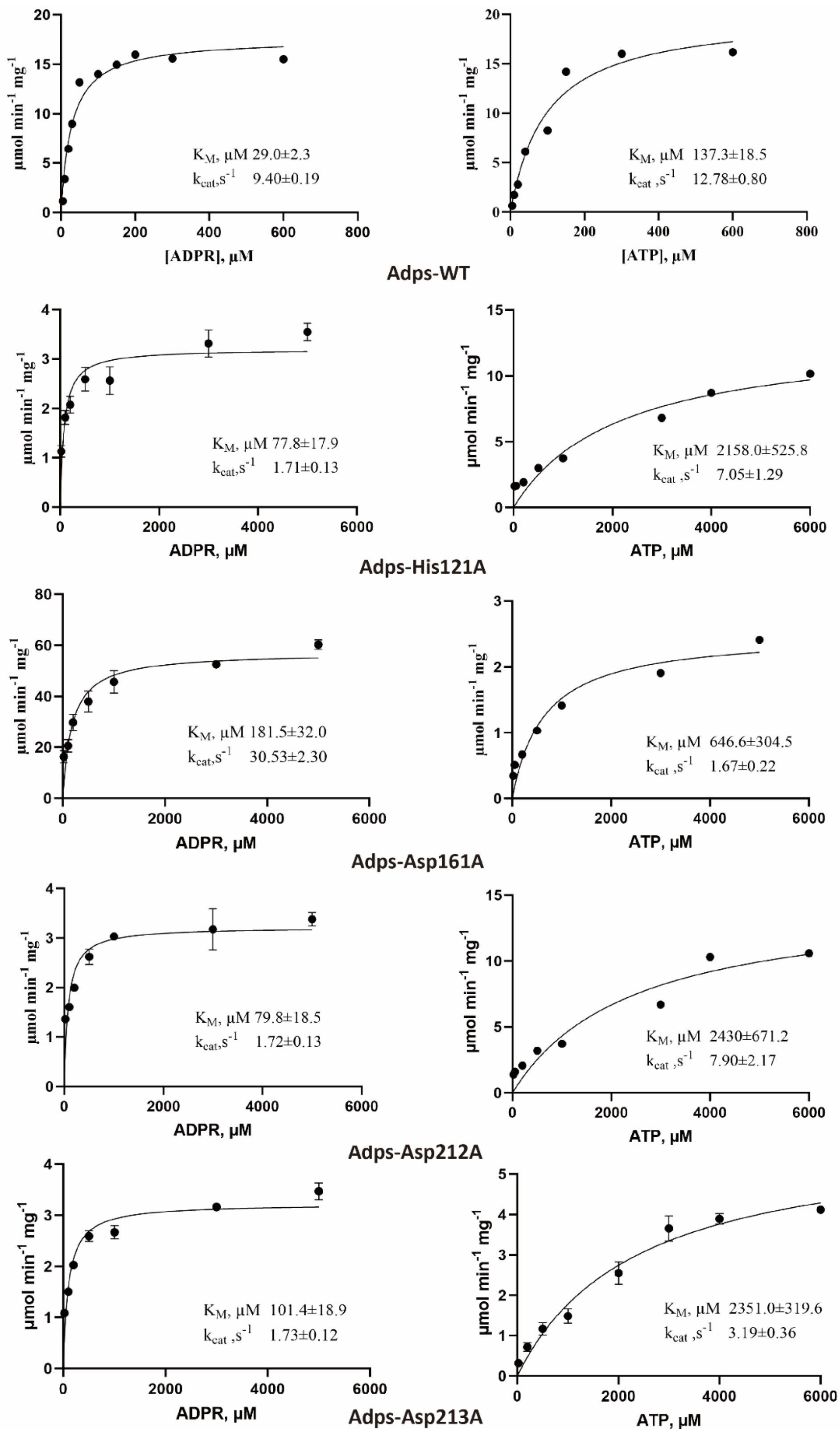

Supplementary Fig. S3. Steady-state kinetic analysis of wild-type Adps and its alanine-substitution variants. Initial reaction velocities of Adps-WT, His121A, Asp161A, Asp212A, and Asp213A were measured at increasing concentrations of ADP-ribose (ADPR; left panels) or ATP (right panels). Solid curves represent nonlinear fits to the Michaelis–Menten equation. The resulting Km and kcat values are shown in each panel. Data are presented as the mean ± SD of three independent experiments (n=3)

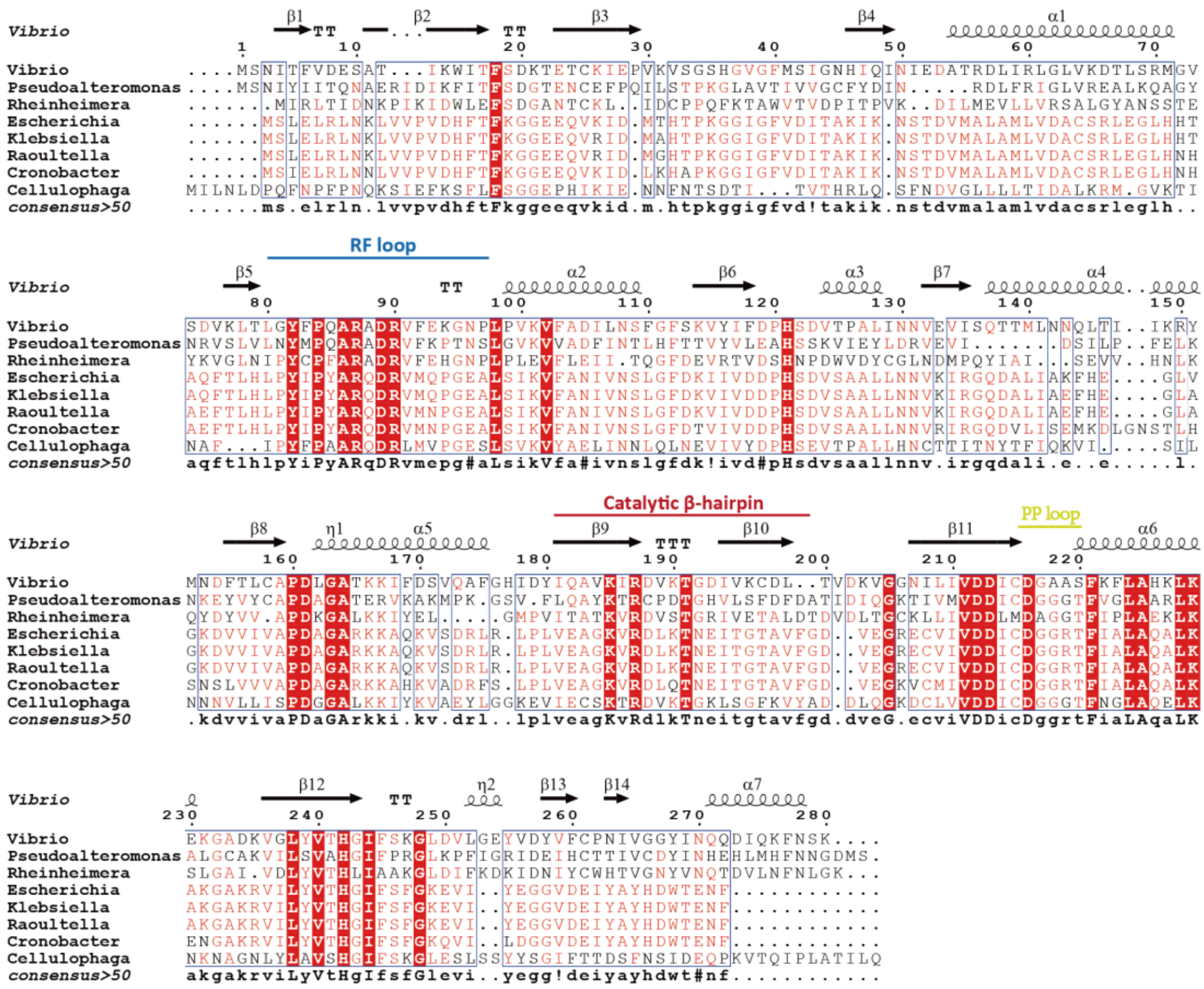

Supplementary Fig. S4. Multiple sequence alignment of representative Adps homologs. Phage-associated Adps sequences were aligned and annotated with secondary-structure elements from Vibrio phage Adps. Conserved motifs involved in substrate recognition and catalysis are highlighted, including the RF loop, catalytic beta-hairpin and PP loop.

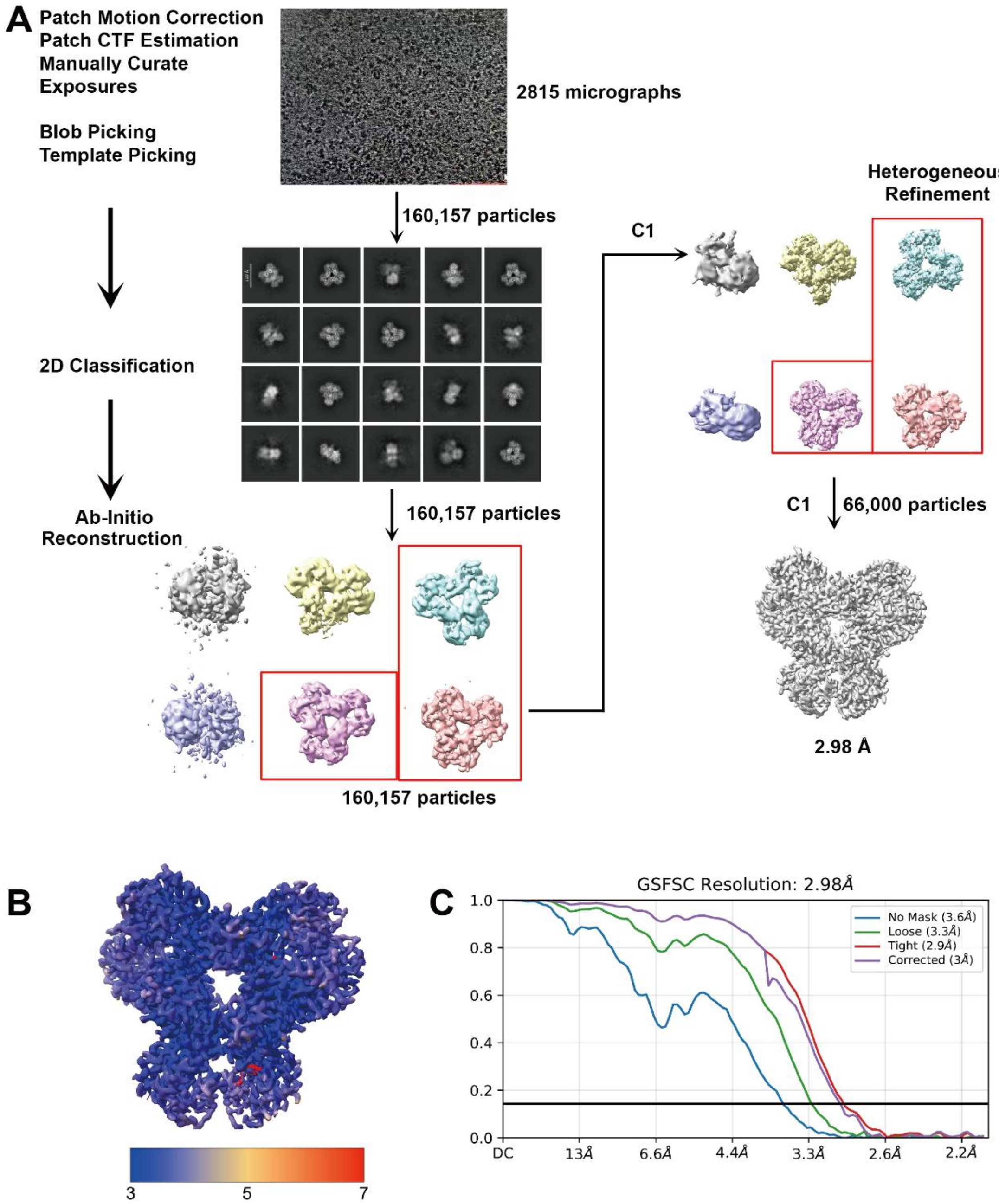

Supplementary Fig. S5. Cryo-EM data processing and validation of the Adps-ADPR-PP-AMP complex. (A) Processing workflow for the product-bound Adps complex, including micrograph selection, particle picking, 2D classification, ab initio reconstruction, heterogeneous refinement and final reconstruction. **(B)** Local-resolution distribution of the final map. **(C)** Gold-standard FSC curves and model-to-map validation for the final reconstruction.

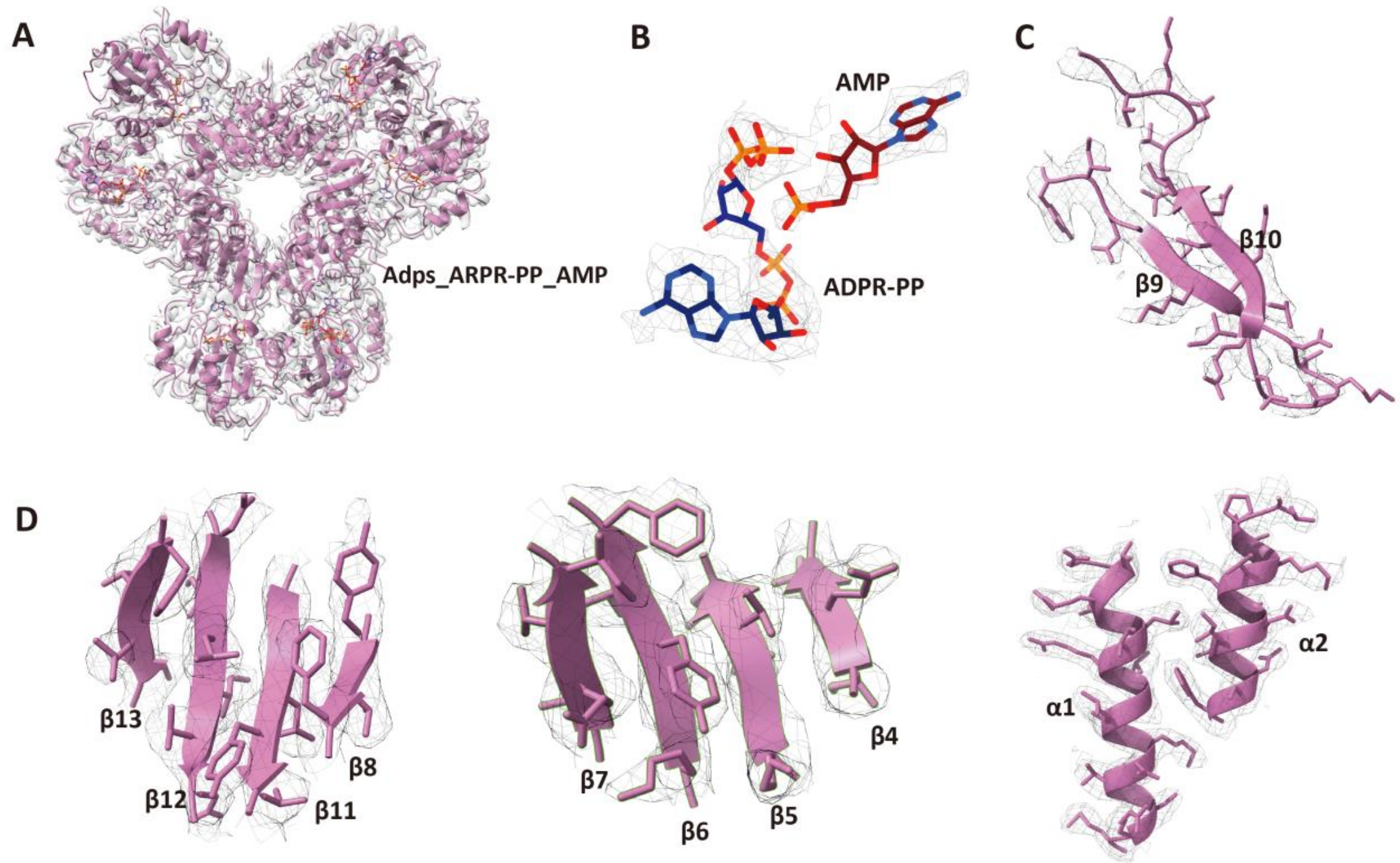

Supplementary Fig. S6. Representative cryo-EM density and model quality for Adps-ADPR-PP-AMP structures. (A) Overall cryo-EM maps with fitted atomic models for the ligand-bound Adps hexamers. **(B)** Representative ligand densities supporting assignment of ADPR/ATP. **(C and D)** Representative local density around selected beta-strands and alpha-helical regions, illustrating the quality of model fitting in both complexes.

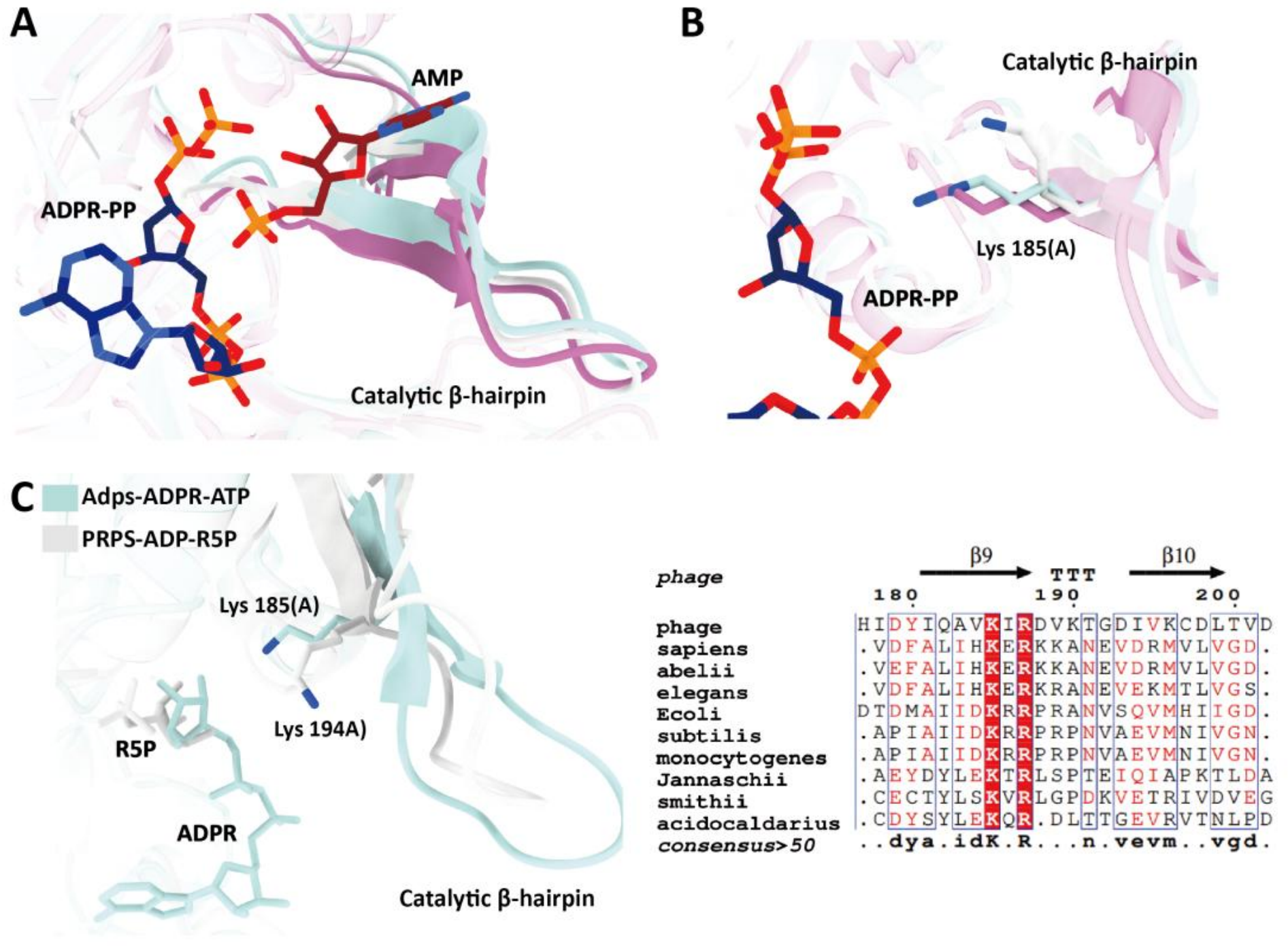

Supplementary Fig. S7. Conformational changes in the catalytic β-hairpin during Adps catalysis. (A) Superposition of apo-Adps (white), the pre-catalytic Adps–ADPR–ATP complex (cyan), and the post-catalytic Adps–ADPR-PP–AMP complex (magenta), highlighting local rearrangements of the catalytic β-hairpin and the altered orientation of AMP following pyrophosphate transfer. ADPR-PP and AMP from the post-catalytic complex

are shown as sticks. **(B)** Close-up view of Lys185 in chain A, showing its progressive inward movement from the apo state through the pre-catalytic state to the post-catalytic state, toward the terminal ribose and newly formed pyrophosphate moiety of ADPR-PP. **(C)** Structural and sequence comparison of the catalytic β-hairpin between Adps and canonical PRPS (PDB:8DBE) enzymes.

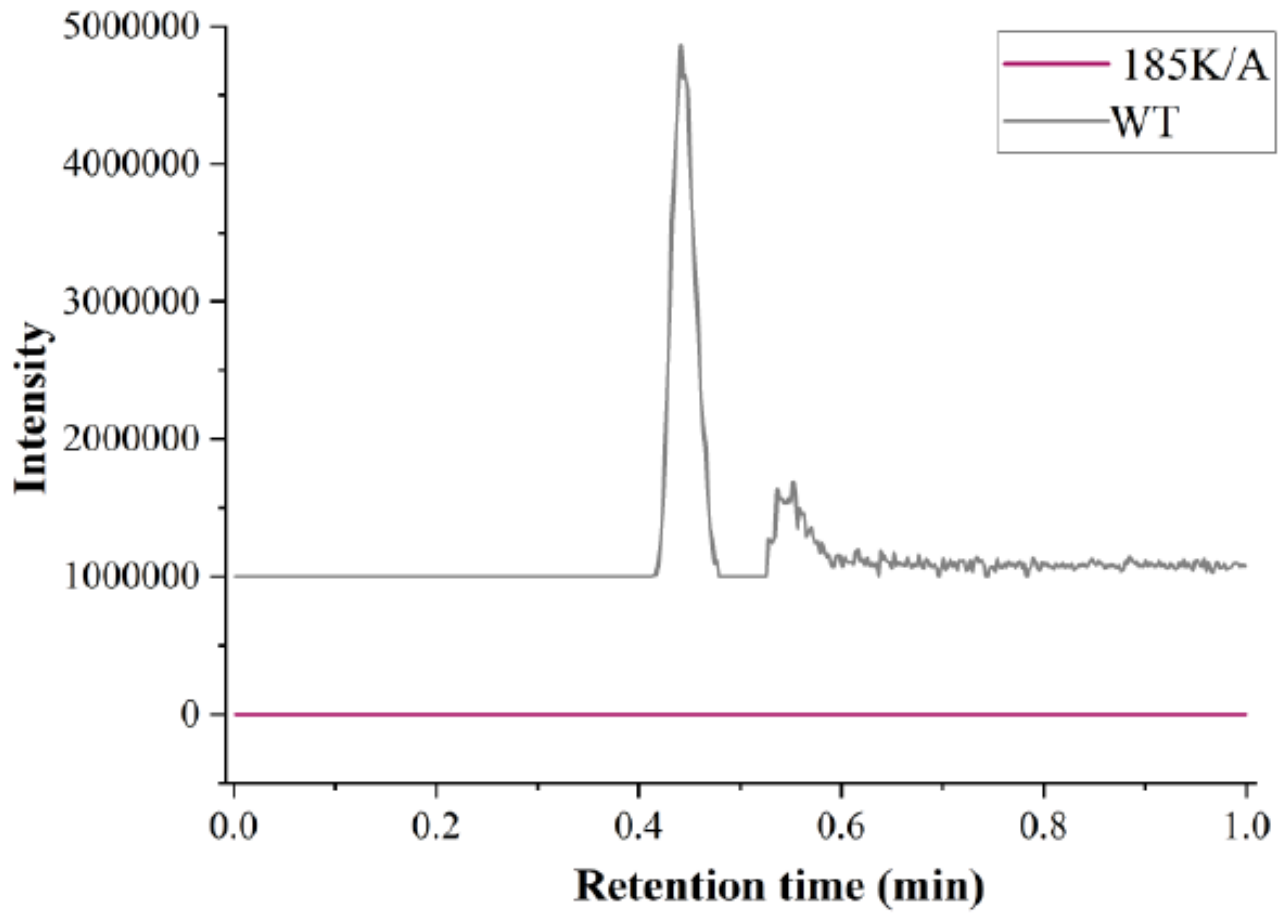

Supplementary Fig. S8. LC-MS analysis showing that K185A abolishes ADPR-PP production. The K185A substitution eliminates detectable ADPR-PP production by Adps. LC–MS chromatograms monitoring ADPR-PP formation in reactions containing wild-type Adps (WT, gray) or the K185A variant (magenta).

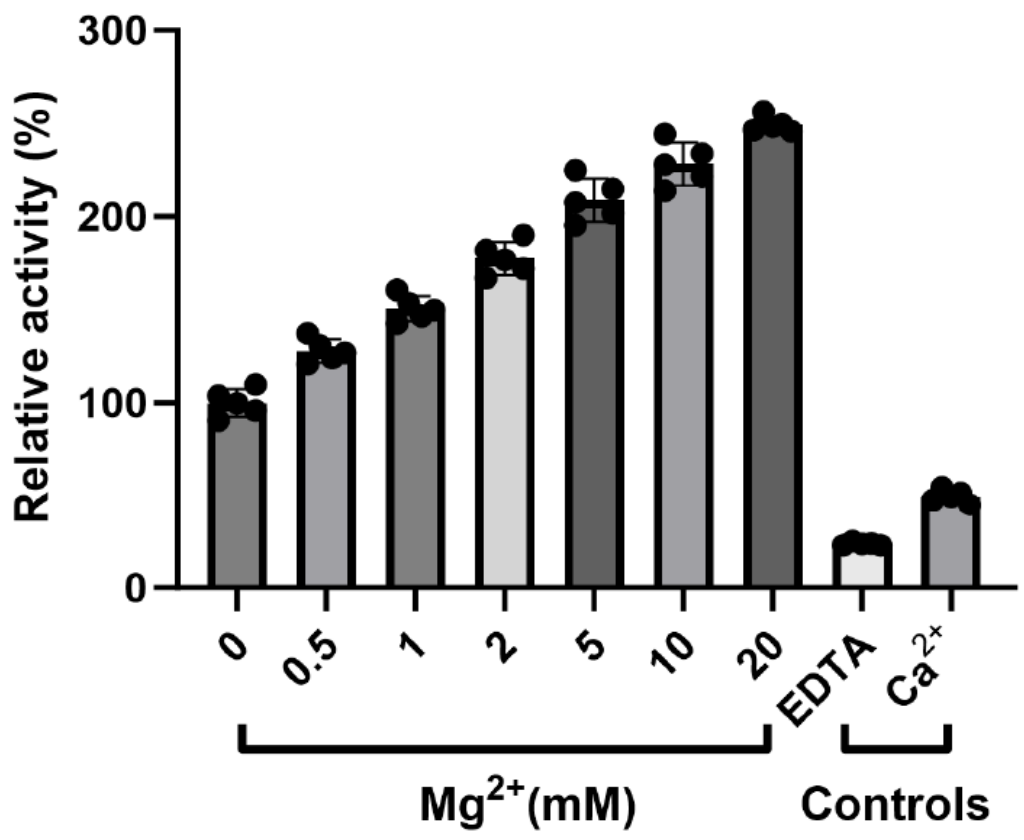

Supplementary Fig. S9. Effect of Mg^{2+} on Adps activity. Adps activity was measured in the presence of the indicated concentrations of $MgCl_2$. Control reactions contained 20 mM EDTA or 20 mM $CaCl_2$. Activity measured in the absence of added $MgCl_2$ was defined as 100%. Bars represent the mean ± SD of three independently prepared reactions, and individual measurements are shown as black circles.

Supplementary Table S1. X-ray crystallography data collection and refinement statistics.

**Dataset**	**Adps**
**Data collection**	
Beamline	BL10U2, SSRF
Wavelength (Å)	0.979183
Space group	*H 3 2*
Cell dimensions	
a, b, c (Å)	105.14, 105.14, 150.45
α, β, γ (°)	90, 90, 120
Resolution range (Å)*	77.9 - 2.61 (2.68 - 2.61)
Completeness (%)	99.2 (99.9)
I/σ(I)	18.5 (1.6)
CC(1/2)	0.995 (0.628)
R_{merge}	0.083 (1.997)
Multiplicity	10.4 (13.6)
**Refinement**	
Resolution (Å)	2.61
No. unique reflections	18746
Rwork/Rfree (%)	21.04/28.30
No. atoms	
Protein	2176
Solvent	42.6
B-factors (Å^2)	
Protein	62.6
Solvent	45.20
R.m.s. deviations	
Bond lengths (Å)	0.009
Bond angles (°)	1.024
Ramachandran plot (%)	
Favored region	96.00
Allowed region	4.00
Outliers	0.00

Supplementary Table S2. Statistics of cryo-EM data of the Adps complex and structure refinement.

**Data collection and processing**		
	**Adps-ADPR -ATP**	**Adps-ADPR-PP-AMP**
Magnification	165K	165K
Voltage (kV)	300	300
Electron exposure (e−/Å2)	50	50
Defocus range (μm)	0.8-2.4	0.8-2.4
Pixel size (Å)	0.71	0.71
Symmetry imposed	D3	C1
Initial particle images (no.)	202,037	160,157
Final particle images (no.)	82,500	66,000
Map resolution (Å)	2.76	2.98
FSC threshold	0.143	0.143
Map resolution range (Å)	2.76-4.5	2.98-4.5
Map sharpening B factor (Å2)	-80	-80
**Model composition**		
Protein residues	282	282
Ligand	ADPR, ATP	ADPR-PP, AMP
***B* factors (Å^2)**		
Protein	20.41/149.31/69.28	41.77 / 153.46 / 86.68
**R.m.s. deviations**		
Bond lengths (Å)	0.003	0.006
Bond angles (°)	0.536	1.057
**Validation**		
molProbity score	1.54	1.83
Clash score	2.17	10.07
Ramachandran plot (%)		
Favored (%)	96.52	95.65
Allowed (%)	3.48	4.29
Outliers (%)	0.00	0.06
